# Polymorphic Control of Solution-Processed Cu_2_SnS_3_ Films with Thiol–Amine Ink Formulation

**DOI:** 10.1021/acs.chemmater.2c01612

**Published:** 2022-09-21

**Authors:** Kristopher M. Koskela, Carlos Mora Perez, Dmitry B. Eremin, Jake M. Evans, Marissa J. Strumolo, Nathan S. Lewis, Oleg V. Prezhdo, Richard L. Brutchey

**Affiliations:** † Department of Chemistry, University of Southern California, Los Angeles, California 90089, United States; ‡ The Bridge@USC, University of Southern California, Los Angeles, California 90089, United States; § Division of Chemistry and Chemical Engineering, California Institute of Technology, Pasadena, California 91125, United States

## Abstract

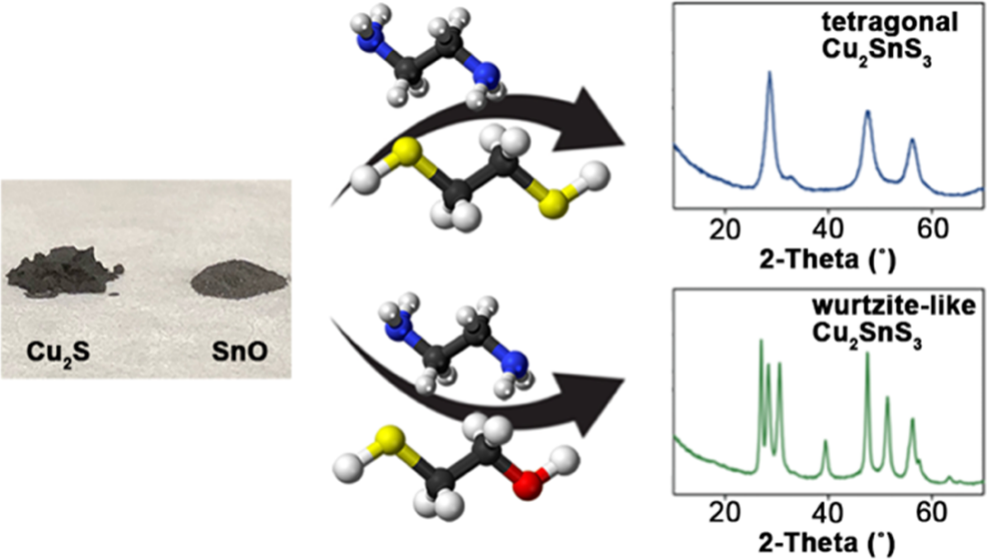

There is increasing demand for tailored molecular inks that produce phase-pure solution-processed semiconductor films. Within the Cu–Sn–S phase space, Cu_2_SnS_3_ belongs to the I_2_–IV–VI_3_ class of semiconductors that crystallizes in several different polymorphs. We report the ability of thiol–amine solvent mixtures to dissolve inexpensive bulk Cu_2_S and SnO precursors to generate free-flowing molecular inks. Upon mild annealing, polymorphic control over phase-pure tetragonal (*I*4̅2*m*) and orthorhombic (*Cmc*2_1_) Cu_2_SnS_3_ films was realized simply by switching the identity of the thiol (i.e., 1,2-ethanedithiol vs 2-mercaptoethanol, respectively). Polymorph control is dictated by differences in the resulting molecular metal–thiolate complexes and their subsequent decomposition profiles, which likely seed distinct Cu_2–*x*_S phases that template the ternary sulfide sublattice. The p-type tetragonal and orthorhombic Cu_2_SnS_3_ films possess similar experimental direct optical band gaps of 0.94 and 0.88 eV, respectively, and strong photoelectrochemical current responses. Understanding how ink formulation dictates polymorph choice should inform the development of other thiol–amine inks for solution-processed films.

## Introduction

Ternary Cu_2_SnS_3_ is a potentially more earth-abundant replacement for current direct band gap semiconductors, such as CdTe and Cu(In,Ga)(S,Se)_2_ (CIGS), while still possessing favorable optoelectronic properties.^1−3^ Cu_2_SnS_3_ belongs to the class of I_2_–IV–VI_3_ semiconductors that possess high optical absorption coefficients (>10^5^ cm^–1^), p-type conductivity, and tunable direct band gaps from 0.9 to 1.8 eV.^3−6^ Several different Cu_2_SnS_3_ syntheses for applications ranging from solar cells,^7−11^ photocatalysts,^12−14^ electrocatalysts,^15,16^ and thermoelectrics^17,18^ have been reported. The I_2_–IV–VI_3_ stoichiometry of Cu_2_SnS_3_ exists in three different crystal structures on the bulk Cu–Sn–S phase diagram, including monoclinic (*Cc*), tetragonal (*I*4̅2*m*), and cubic (*F*4̅3*m*).^19,20^ The monoclinic structure is isomorphic to the Cu_2_SiS_3_ structure type.^20^ Among the disordered structure types, the cubic zinc blende polymorph has *F*4̅3*m* symmetry and the tetragonal stannite polymorph (supercell of zinc blende) has *I*4̅2*m* symmetry.^20^ Cu_2_SnS_3_ has also been reported to form a hexagonal wurtzite (**P**6_3_**mc**) phase in colloidal nanocrystals, but this purported polymorph does not exist on the bulk Cu–Sn–S bulk phase diagram.^1,3,14,19,21^

The structure of a material directly affects its electronic and optical properties.^22,23^ For example, metastable wurtzite-like Cu_2_ZnSn(S_1–*x*_Se_*x*_)_4_ nanocrystals have wider band gap tunability compared to the thermodynamic kesterite phase in the same compositional range.^24^ While colloidal nanocrystal syntheses allow for the formation of metastable phases on bulk phase diagrams, or the isolation of phases previously unknown in bulk,^25^ the utilization of these nanocrystals in functional devices is generally hampered by the insulating nature of the organic surface ligands. Methods used to remove ligands for devices involve complex ligand exchanges or thermally decomposing the ligands, which can leave large carbonaceous impurities in the semiconductor layer and at interfaces.^26,27^ No direct solution deposition of metastable Cu_2_SnS_3_ thin films using molecular inks has been previously reported.

In 2013, our group developed a simple and versatile “alkahest” solvent system consisting of a short chain thiol (e.g., 1,2-ethanedithiol, mercaptoethanol, etc.) and an amine (e.g., 1,2-ethylenediamine) that dissolves over 100 bulk materials, including oxides, chalcogenides, and zero-valent metals. This dissolution process yields molecular inks amenable to solution processing; phase-pure metal chalcogenide thin films can be recovered upon solution processing and mild heating.^28,29^ The alkahest solvent system has been leveraged to solution process high-efficiency solar cells,^30,31^ electrocatalysts,^32,33^ thermoelectrics,^34,35^ and a few reports of nanostructured devices, such as tremella-like SnS_2_ and Sb_2_Se_3_ nanowires.^36,37^ While compositional control in multinary chalcogenide semiconductors can be achieved by tuning the solute formulation of alkahest-derived inks,^38,39^ polymorphic phase control has yet to be demonstrated. Herein, we report phase control over two different Cu_2_SnS_3_ structural polymorphs simply by tuning the ink formulation (i.e., thiol choice of 1,2-ethanedithiol or mercaptoethanol). We propose an alternate orthorhombic structure (*Cmc*2_1_) for the metastable Cu_2_SnS_3_ phase, with a hexagonally close-packed S^2–^ sublattice that is isostructural with orthorhombic Ag_2_GeS_3_.^40^ To the best of our knowledge, this is the first example where the metastable Cu_2_SnS_3_ polymorph has been observed outside the context of colloidal nanocrystals. The preparation of the two phase-pure Cu_2_SnS_3_ polymorphs using alkahest inks, and their characterization, are discussed in detail. Density functional theory (DFT) calculations were carried out within the PBE & HSE06 level of theory to compare the optoelectronic properties of the thermodynamically stable monoclinic structure to the proposed metastable orthorhombic polymorph of Cu_2_SnS_3_.

## Experimental Section

### General Considerations

All materials were used as received. 1,2-Ethylenediamine (en, 99.5%), 2-mercaptoethanol (merc, 99%), and copper(I) sulfide (Cu_2_S, 99.99%) were purchased from Sigma-Aldrich. 1,2-Ethanedithiol (EDT, 98+%) and tin(II) oxide (SnO, 99%) were purchased from Alfa Aesar.

### Ink Formulations and Processing

To generate the ink for tetragonal Cu_2_SnS_3_, 9.95 mg (0.063 mmol) of Cu_2_S and 8.42 mg (0.063 mmol) of SnO were added to 0.2 mL of EDT and 0.8 mL of en and allowed to stir for 2 h at 30 °C. Full dissolution is observed to occur within minutes. If the EDT/en (1:4 vol/vol) solvent mixture solidifies, gentle heating will resolubilize the solution. To generate the ink for orthorhombic Cu_2_SnS_3_, 9.95 mg (0.063 mmol) of Cu_2_S and 8.42 mg (0.063 mmol) of SnO were added separately to 0.1 mL of merc and 0.4 mL of en and allowed to dissolve for up to 2 h at 30 °C with stirring before finally being combined after full dissolution right before deposition and annealing.

Inks to produce the tetragonal and orthorhombic polymorphs of Cu_2_SnS_3_ were drop-cast onto fluorine-doped tin oxide (FTO), Si, or borosilicate glass substrates that were cleaned by sequential sonication in methanol, acetone, and isopropyl alcohol for 15 min each before being blown dry using pressurized nitrogen gas. Before deposition, the substrates were ozone cleaned for 15 min. Finally, 20 μL of ink was deposited onto 1 × 1 cm^2^ precut substrates, and the tip of the pipette was used to spread the ink across the full substrate without allowing the tip of the syringe to touch the surface of the substrate. Films were annealed identically under flowing nitrogen to 330 °C (ca. 15 °C min^–1^ ramp rate) and held for 10 min, and then allowed to cool naturally to room temperature.

### Organic Content Determination

Thermal gravimetric analysis (TGA) was performed on a TA Instruments TGA Q50 instrument, and samples were run in an alumina crucible under a flowing nitrogen atmosphere with a heating rate of 5 °C min^–1^. The TGA samples were prepared by drying the ink in an alumina crucible to 100 °C under a flowing nitrogen atmosphere in an aluminum annealing chamber prior to TGA analysis to avoid excessive corrosion of the thermocouple in the TGA. Fourier transform infrared spectroscopy (FT-IR) spectra were collected on an Agilent Cary 630 spectrometer by diamond attenuated total reflection (ATR). The samples were prepared by drop casting the inks onto glass substrates and drying to 100 °C before annealing to 330 °C under a flowing stream of nitrogen and transferring the Cu_2_SnS_3_ to the crystal.

### Structural and Optical Characterization

Powder X-ray diffraction (XRD) patterns were collected using a Rigaku Ultima IV diffractometer operated at 44 mA and 40 kV, in the 2θ range of 10–70° using Cu Kα radiation (λ = 1.5406 Å). For powder diffraction studies, inks were drop-cast on a glass substrate and dried to 350 °C in an aluminum annealing chamber under flowing nitrogen. The powders were removed from the glass substrate and ground in an agate mortar. For structural refinements, the step size and collection time were 0.01° and 3 s step^–1^. All patterns were recorded under ambient conditions. Rietveld refinements were carried out using the General Structure Analysis System II (GSAS-2) software package. The following parameters were refined: (1) scale factor, (2) background (modeled using a shifted Chebyshev polynomial function), (3) peak shape, (4) lattice constants, (5) fractional atomic coordinates of the Cu, Sn, and S atoms constrained by the site symmetry, (6) preferred orientation using a spherical harmonic model, and (7) isotropic thermal parameters for each chemical species. The *R*_wp_ and χ^2^ indicators were employed to assess the quality of the refined structural models. Diffuse reflectance UV–vis–NIR spectroscopy was performed on a PerkinElmer Lambda 950 equipped with a 150 mm integrating sphere; 12 mg of powdered Cu_2_SnS_3_ sample (tetragonal or orthorhombic polymorph) was mixed with 350 mg of BaO in a mortar and pestle and placed in a solid sample holder. Transmittance UV–vis–NIR spectroscopy was performed by placing a drop-cast film in front of the detector and recording a transmittance spectrum from 300 to 900 nm. Scanning electron microscopy/energy dispersive X-ray spectroscopy (SEM–EDS) was performed using an FEI Helios G4 P-FIB at 20 kV. Top surface micrographs were acquired via SEM using a beam current of 0.8 nA and an accelerating voltage of 5 kV. Raman spectra were conducted on samples deposited on Si substrates annealed to 330 °C. Spectra were recorded for 1 min using an average of three scans using a Horiba XploRA confocal Raman microscope with 532 nm excitation. The Raman microscope was covered with a black tarp to reduce ambient light exposure. X-ray photoelectron spectroscopy (XPS) was performed using a Kratos Axis Ultra X-ray photoelectron spectrometer with a monochromatic aluminum anode (1486.6 eV). An operating current of 5 mA and voltage of 12 kV with a step size of 0.1 eV and a pass energy of 20 eV was used to acquire 20 high-resolution scans for each element. An operating current of 5 mA and voltage of 12 kV with a step size of 1 eV and pass energy of 80 eV was used to acquire five survey scans for each sample. Pressure in the analysis chamber was <1 × 10^–9^ Torr. XPS was performed on Cu_2_SnS_3_ thin films deposited on Si substrates. Inductively coupled plasma–mass spectrometry (ICP-MS) was conducted with a third-party service (Galbraith Laboratories, Knoxville, TN).

### Molecular Solute Identification

Electrospray ionization–mass spectrometry (ESI-MS) samples were prepared from fully dissolved solutions of Cu_2_S (20 mg mL^–1^, 1:4 (vol/vol) EDT/en or merc/en) diluted with DMSO to ca. 1 μM. Solutions were mixed right before injection into the MS instrument to avoid possible decomposition products. Samples were injected through the main nebulizer using a syringe pump fitted with a 500 μL Hamilton syringe (1750RN) at 5 μL min^–1^ flow rate. Mass spectra were measured using Agilent 6545 qToF instrument equipped with a dual AJS electrospray ionization source operating in negative ion mode with the following ionization parameters: capillary voltage 3.5 kV, nozzle voltage 0.0 kV, nitrogen was applied as a nebulizer gas 35 psi, sheath gas 12 L min^–1^, 275 °C, dry gas 10 L min^–1^, 300 °C, and collision gas. For external calibration and tuning, a low-concentration tuning mix solution by Agilent Technologies was utilized at 10:1 for further dilution. Spectra were recorded in *m*/*z* 50–2000 range. All of the mass spectra were recorded at 1 Hz.

### Photoelectrochemical Measurements

Photoelectrochemical measurements were performed on FTO substrates (Sigma-Aldrich) with a conductivity of ca. 7 Ω sq^–1^ that were cut into 0.5 × 2.5 cm^2^ pieces and cleaned as described above. Kapton tape was used to mask off a portion of the substrate for electrode contact. Photoelectrochemical responses for both polymorphs of Cu_2_SnS_3_ were performed using a BASi Epsilon-EC potentiostat. A 3-neck flask was used with a Pt-wire counter electrode and a Pt-wire pseudoreference electrode. The working electrodes were tetragonal and orthorhombic Cu_2_SnS_3_ films drop-cast on FTO-coated glass and annealed to 330 °C. The tetragonal Cu_2_SnS_3_ electrode was further annealed at 550 °C to improve the rigidity of the working electrode and prevent delamination during measurements. An aqueous 0.1 M Na_2_S/0.01 M sulfur electrolyte was made from nitrogen-sparged deionized water. For photoelectrochemical experiments, a standard laboratory white light placed ca. 15 cm from the samples was used to illuminate the working electrode. The total illumination areas of the tetragonal and orthorhombic Cu_2_SnS_3_ working electrodes were ∼0.75 cm^2^.

### Computational Methodology

Density functional theory (DFT)^41−43^ with the projector-augmented-wave (PAW) potentials^44,45^ and the Perdew–Burke–Ernzehof (PBE) functional under the generalized gradient approximation^46,47^ was employed in geometry optimization calculations. The optimized structures can be seen in Figure S1. Due to the known tendency of the PBE functional to strongly underestimate the band gap because of the self-interaction error, the more accurate and computationally expensive Heyd–Scuseria–Ernzehof (HSE06)^48−51^ hybrid functional was used to further evaluate the total energy and electronic structure. All calculations were performed within the Vienna Ab-initio Simulation Package (VASP).^52−55^ The PAW PBE versions included in the POTCAR files for each species were PAW_PBE Cu 22Jun2005, PAW_PBE S 06Sep2000, and PAW_PBE Sn 08Apr2002. During the geometry optimization with PBE, we utilized a large plane wave basis energy cutoff (ENCUT) of 520 eV. An 8 × 8 × 8 Γ centered *k*-point mesh was used to sample the Brillouin zone. Due to the high computation expense of the HSE06 calculations, the ENCUT was lowered to 400 eV. Further, to accurately probe the electronic structure with the HSE06 functional, we set the *k*-point mesh along its high-symmetry *k*-paths: Z–G–Y–A–B–D–E–C and Γ–Z–T–Y–S–X–U–R, for the monoclinic and orthorhombic polymorphs, respectively.

## Results and Discussion

### Ink Formulation and Conversion

To formulate a typical ink to yield tetragonal Cu_2_SnS_3_, bulk powders of Cu_2_S and SnO were mixed in a 1:1 (mol/mol) stoichiometric ratio in EDT/en (1:4 vol/vol) with an overall concentration of ca. 20 mg mL^–1^. The bulk precursors may be dissolved together or separately to return to the tetragonal phase. Bulk Cu_2_S and SnO powders both have overall solubility limits of 10–15 wt % in EDT/en (1:4 vol/vol) mixtures under ambient conditions (1 atm, 25 °C). The inks were stirred at 30 °C for 2 h to yield a free-flowing, optically clear orange-brown ink free of scattering. The ink was stable for multiple days under inert atmosphere conditions, as indicated by the lack of color change and solid precipitates. To formulate a typical ink to yield orthorhombic Cu_2_SnS_3_, bulk powders of Cu_2_S and SnO were separately dissolved in a 1:1 (mol/mol) ratio in a mixture of merc/en (1:4 vol/vol) with an overall concentration of ca. 20 mg mL^–1^ in each ink. The bulk Cu_2_S and SnO powders have solubility limits of 10–15 and 5–10 wt %, respectively, in merc/en (1:4 vol/vol) mixtures under ambient conditions (1 atm, 25 °C). The SnO ink in merc/en was stirred at 30 °C for 2 h to yield a free-flowing, optically clear, and colorless ink free of scattering. The Cu_2_S ink in merc/en was a faint light brown color, optically clear, and free of scattering after dissolution under the same conditions. As described above, both Cu_2_S and SnO inks were stable for multiple days under inert atmosphere conditions. Right before annealing, the two Cu_2_S and SnO inks in merc/en were mixed to form an almost colorless and optically clear ink. When the bulk precursors were codissolved in merc/en, mixed phase products were recovered upon annealing. While the bulk Cu_2_S and SnO precursors returned phase-pure tetragonal and orthorhombic phases (vide infra), using different combinations of oxides and sulfides (i.e., CuO, CuS, Cu_2_O, SnS, SnS_2_) also returned the tetragonal and orthorhombic polymorphs when using EDT/en and merc/en solvents, respectively. To test the viability of thiol–amine solutions to produce metal chalcogenides at scale, inks made at 10× volumetric scale also produced the same phase-pure tetragonal and orthorhombic phases of Cu_2_SnS_3_ when annealed at 330 °C for 10 min.

Thermogravimetric analysis (TGA) was employed to determine the endpoint of organic volatilization and molecular solute decomposition for dried inks from both EDT/en and merc/en solvent combinations (Figure S2). Organic mass loss begins ca. 120 °C and mass loss ends at temperatures <350 °C for both inks. The strongest IR bands of the dried inks belong to the thiol (∼675 cm^–1^ ν(C–S) stretch, ∼1430 cm^–1^ δ(CH_2_) bend);^29,56,57^ however, upon annealing the dried inks to 330 °C, a complete loss of IR bands is observed that corroborates the decomposition temperature measured by TGA (Figure S3).

### Structural Characterization

**Figure 1:**
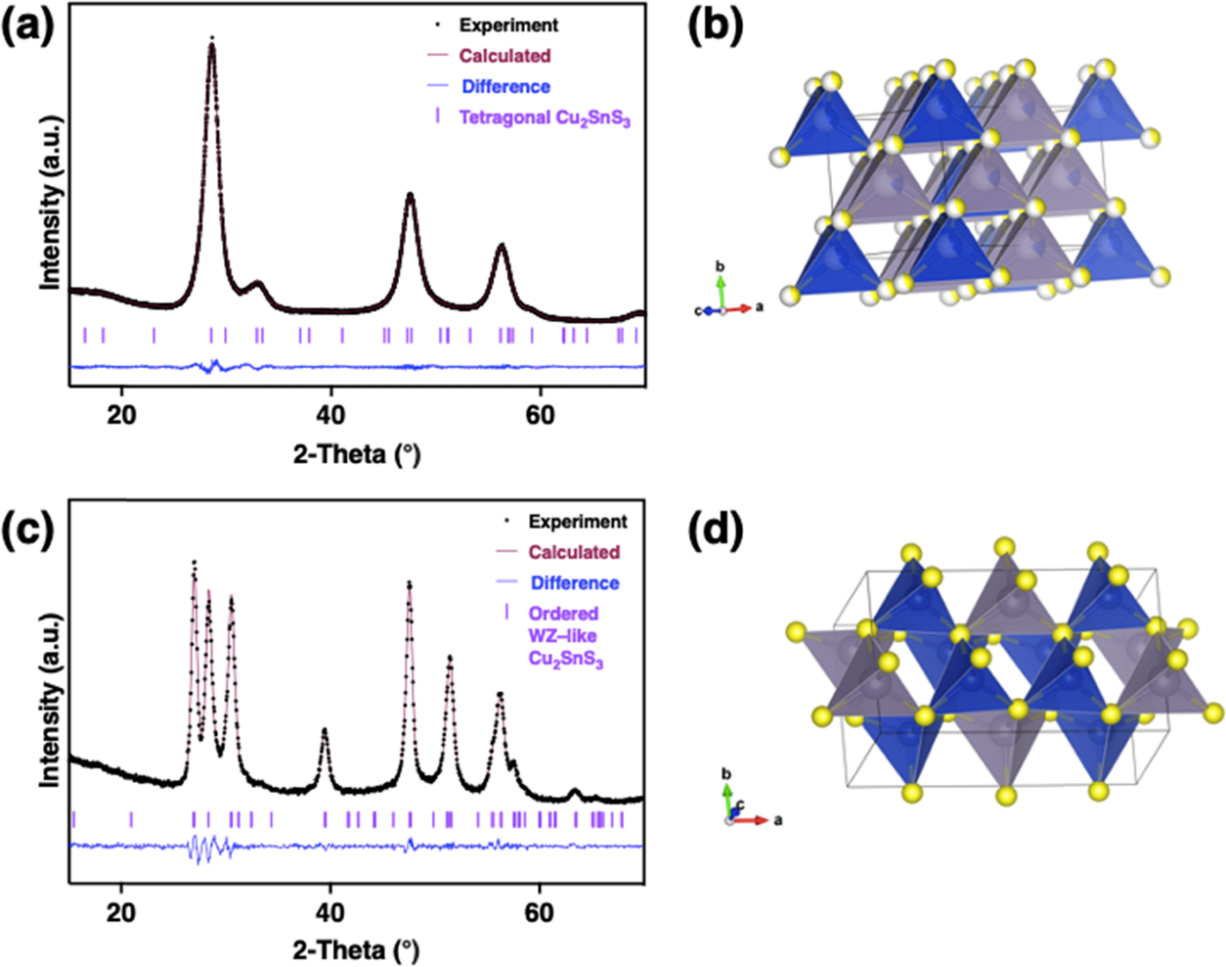
(a) Rietveld refinement of the XRD data corresponding to Cu_2_SnS_3_ resulting from the EDT/en ink, confirming that the tetragonal *I*4̅2*m* unit cell is an appropriate structural model for this polymorph (χ^2^ 1.69, w*R* 2.29%; *a* = 5.43 Å, *c* = 10.69 Å, *V* = 315.19 Å^3^). (λ = 1.5406 Å) (b) Structure of disordered tetragonal Cu_2_SnS_3_. (c) Rietveld refinement of the XRD data corresponding to Cu_2_SnS_3_ resulting from the merc/en ink, confirming that the orthorhombic *Cmc*2_1_ unit cell is an appropriate structural model for the metastable polymorph (χ^2^ 2.18, w*R* 4.02%, *a* = 11.46 Å, *b* = 6.63 Å, *c* = 6.32 Å, *V* = 479.95 Å^3^). (λ = 1.5406 Å) (d) Structure of ordered orthorhombic Cu_2_SnS_3_. Sulfur atoms are yellow, tin atoms are silver, and copper atoms are blue.

The resulting dark gray materials after solution deposition and annealing to 330 °C were confirmed to be phase-pure tetragonal and orthorhombic Cu_2_SnS_3_ by powder X-ray diffraction from the EDT/en and merc/en inks, respectively. Rietveld refinements were employed to determine the proper space group for each phase. For the Cu_2_SnS_3_ resulting from the EDT/en ink, three phases are possible candidates (i.e., monoclinic, tetragonal, cubic), and their diffraction patterns only differ slightly by minor reflections. Representations of the crystal structures used for refinements are supplied in the Supporting Information. To assess the phase of Cu_2_SnS_3_ resulting from the EDT/en ink, powder XRD data sets were refined against these monoclinic (*Cc*), tetragonal (*I*4̅2*m*), and cubic (*F*4̅3*m*) crystal structures. In the first refinement, the monoclinic phase failed to converge. In the second refinement, the disordered tetragonal phase led to a refinement with a reduced χ^2^ of 1.69 and a w*R* of 2.29% (Figure 1a,b, Table S1). The Rietveld refinement to the tetragonal structure returned lattice constants of *a* = 5.4267(6) Å and *c* = 10.6869(3) Å with a unit cell volume of *V* = 314.72(8) Å^3^, which match well to previously reported values for bulk tetragonal Cu_2_SnS_3_ (*a* = 5.41 Å, *c* = 10.82 Å, *V* = 316.68 Å).^58^ In the third refinement, we simulated a disordered cubic zinc blende structure in which the Cu and Sn atoms are randomly distributed over the Zn site as there are no published zinc blende Cu_2_SnS_3_ structures in the ICSD; this refinement returned a slightly worse reduced χ^2^ of 3.56 and a w*R* of 3.32% (Figure S4).

The Cu_2_SnS_3_ resulting from the merc/en ink yields a distinctly wurtzite-like diffraction pattern. This polymorph has previously only been observed in colloidal nanocrystals and has been assumed to adopt a hexagonal wurtzite (**P**6_3_**mc**) structure that does not exist on the Cu–Sn–S bulk phase diagram.^1,3,14,19,21^ The disordered wurtzite polymorph derives from the ZnS wurtzite structure, where Cu^+^ and Sn^4+^ randomly occupy the Zn site with a 2:1 ratio, respectively. However, there are no known published structures for wurtzite Cu_2_SnS_3_. A simulated wurtzite cell with Cu and Sn randomly distributed (2:1 ratio, respectively) on the Zn site, while preserving the sulfur positions, returned a reduced χ^2^ of 3.05 and a w*R* of 4.75% (Figure S5). However, tetrahedrally bonded ternary I–III–VI_2_ sulfides with hexagonal S^2–^ sublattices, such as wurtzite-like CuInS_2_ and AgInS_2_, are known to possess cation ordering and not adopt a true disordered wurtzite structure.^59,60^ This led us to consider whether there are any known I_2_–IV–VI_3_ materials with hexagonal S^2–^ sublattices, which revealed the orthorhombic polymorph (*Cmc*2_1_) of Ag_2_GeS_3_. This structure is like the wurtzite structure type with a hexagonal close-packed S^2–^ sublattice, but with the major distinction being that there is an ordering of the Ag^+^ and Ge^4+^ cations. We simulated the orthorhombic structure type by replacing the Ag^+^ and Ge^4+^ cations with Cu^+^ and Sn^4+^ and allowed the cell and atoms to relax using the GGA PBE exchange-correlation functional with forces converged to 3 meV Å^–1^. This structure type resulted in an improved refinement with a reduced χ^2^ of 2.18 and w*R* of 4.02% (Figure 1c,d and Table S2). The refinement returned lattice parameters of *a* = 11.4569(4) Å, *b* = 6.6268(9) Å, *c* = 6.3215(7) Å, and *V* = 479.95(8) Å^3^, matching quite well to the simulated crystal structure (*a* = 11.45 Å, *b* = 6.62 Å, *c* = 6.34, *V* = 480.56 Å^3^). This suggests a potential for cation ordering in our wurtzite-like Cu_2_SnS_3_ polymorph due to the fitting of minor reflections and intensity mismatch of the difference curve at 2θ = 47.9°. In our search for an ordered structure, we simulated several other possibilities for an ordered wurtzite-like structure. The simplest ordered cell we considered was the wurtzite ZnS structure expanded to a 1 × 1 × 3 supercell, where three ordering pattern iterations were possible. Expanding the supercell to 1 × 2 × 3 led to 45 ordering iterations. The ordered orthorhombic 1 × 1 × 1 cell led to 77 different ordering iterations with similar minor reflections (only differing in reflection intensities), leading to statistically similar refinements. The resulting orthorhombic Cu_2_SnS_3_ films remained in this metastable polymorph for over 4 months at room temperature. “Wurtzite” Cu_2_SnS_3_ has been previously reported to undergo a phase transition to the zinc blende polymorph at elevated temperatures (>500 °C).^3^ Our orthorhombic polymorph was similarly annealed at 550 °C and experimentally confirmed to relax to either the cubic zinc blende or tetragonal polymorph (Figure S6).

The Rietveld refinements for the tetragonal and orthorhombic Cu_2_SnS_3_ polymorphs were not improved with the addition of any binary Cu_2–*x*_S, SnS, or SnS_2_ phases, suggesting that both materials are phase pure. Average grain sizes of 11.0 and 68.0 nm for the tetragonal and orthorhombic polymorphs, respectively, were extracted from refinements. Raman spectroscopy data corroborated the phase purity of the solution-processed tetragonal and orthorhombic Cu_2_SnS_3_ materials. For the tetragonal polymorph, a Raman active mode at 324 cm^–1^ matches well with spectra previously reported for tetragonal Cu_2_SnS_3_ (Figure S7a).^61^ The orthorhombic polymorph has Raman active modes at 291 and 314 cm^–1^ that match well to previously reported “wurtzite” Cu_2_SnS_3_ (Figure S7b).^3^ Neither have Raman active modes from potential binary impurities, such as Cu_2–*x*_S, SnS, or SnS_2_.^62^

The broadness of the XRD peaks and small grain sizes indicate potential nanostructuring of the resulting solution-processed Cu_2_SnS_3_ films. Scanning electron microscopy (SEM) images of drop-cast tetragonal and orthorhombic Cu_2_SnS_3_ films on Si substrates confirm the presence of nanostructured grains (Figures S8 and S9). While the metastable phase has only been observed as ligand-stabilized colloidal nanocrystals, the SEM images coupled with the FT-IR spectra prove the solution deposition of ligand-less orthorhombic Cu_2_SnS_3_ directly from molecular thiol–amine solutions. ICP-MS was used to assess the average elemental composition of our drop-cast films. Elemental compositions of Cu_1.85_Sn_0.92_S_3.00_ (Cu/(Cu + Sn) = 0.67) for the tetragonal polymorph and Cu_1.98_Sn_1.02_S_3.00_ (Cu/(Cu + Sn) = 0.66) for the orthorhombic polymorph were obtained. These values are within the range of previously reported experimental stoichiometries for tetragonal and “wurtzite” samples, as Cu_2_SnS_3_ is known to possess wide compositional variations with the Cu/(Cu + Sn) ratio ranging from 0.26 to 0.72 for the tetragonal polymorph and 0.49–0.81 for the “wurtzite” polymorph.^1^

X-ray photoelectron spectroscopy was used to gain insights into the valence states of the tetragonal and orthorhombic polymorphs. Survey scans of drop-cast tetragonal and orthorhombic Cu_2_SnS_3_ on Si substrates that had been exposed to atmospheric oxygen are provided in the Supporting Information (Figures S10 and S11). High-resolution spectra of the Cu 2p, Sn 3d, and S 2p regions of the tetragonal Cu_2_SnS_3_ are given in Figure S12, and the corresponding peak fittings are given in Table S3. The Cu 2p region can be fit with a single set of doublets at 952.0 and 932.2 eV with a splitting of 19.8 eV, consistent with an assignment of Cu^+^ in tetragonal Cu_2_SnS_3_. The Sn 3d region can be fit with two sets of doublets, each with a splitting of 8.4 eV. The lower binding energy peaks at 494.8 and 486.4 eV (FWHM = 0.78 eV) match well to Sn^4+^–S.^61^ The smaller, higher binding energy doublet at 495.2 and 486.8 eV (FWHM = 1.75 eV) most likely corresponds to amorphous surface oxide or other nonstoichiometric tin oxides (SnO_*x*_) due to Sn–O having a higher binding energy than corresponding Sn–S species, as well as amorphous oxides displaying larger FWHM values.^63^ This is reasonable to expect as our materials were exposed to atmospheric oxygen several times during processing and travel for measurements, consistent with the presence of an O 1s peak in the XPS survey scan. The S 2p spectrum is fit well by a doublet with a splitting of 1.2 eV and S 2p_3/2_ peak centered at 161.4 eV. This is consistent with an assignment of an S^2–^ metal sulfide.^39,63^ A doublet at higher binding energies corresponds to oxidized surface SO_*x*_ species. Thus, XPS analysis confirms the expected formal valence states of the tetragonal polymorph to be (Cu^+^)_2_(Sn^4+^)(S^2–^)_3_. High-resolution spectra of the Cu 2p, Sn 3d, and S 2p regions of the orthorhombic phase are given in Figure S13, and the corresponding peak fittings are given in Table S4. The Cu 2p region can also be fit with a single set of doublets, indicating a single Cu^+^ environment, matching prior literature.^1,3,39,61,64^ The Sn 3d spectrum was fit with two sets of doublets corresponding to Sn^4+^ in a sulfide environment, but the peaks corresponding to surface oxide are much larger relative to the Sn–S peaks when compared to the tetragonal phase.^1,3^ The S 2p region is also fit with two sets of doublets, each with a splitting of 1.2 eV, which matches well with prior literature for an S^2–^ metal sulfide environment in addition to oxidized surface SO_*x*_ species.^1,3,39,61,64^

### Electronic Structure Calculations of Cu_2_SnS_3_ Polymorphs

DFT calculations indicate that the orthorhombic polymorph of Cu_2_SnS_3_ is metastable, with a hull energy of 7.6 meV atom^–1^ above the lowest-energy thermodynamically stable monoclinic polymorph of Cu_2_SnS_3_. In principle, metastable materials with predicted energies of ∼0.1 eV atom^–1^ above the ground state are synthesizable under appropriate conditions, depending on the chemical composition.^65^ The low-lying metastability of our wurtzite-like orthorhombic polymorph is not unprecedented as the median energy above the ground state for metastable ternary polymorphs is 6.9 meV atom^–1^, irrespective of composition.^66^

**Figure 2:**
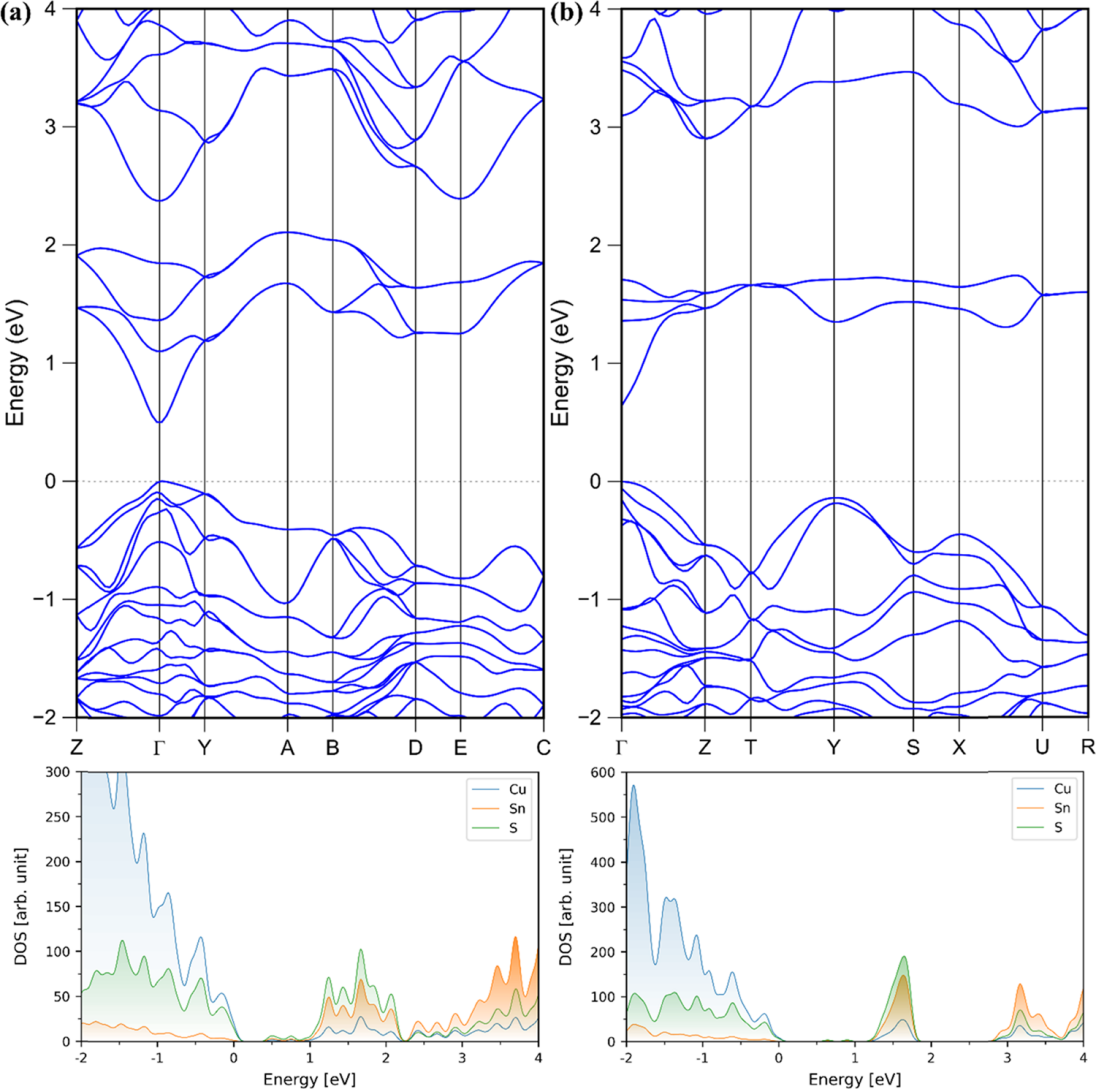
Electronic band structure and density of states (DOS) calculated with the HSE06 functional for (a) monoclinic and (b) orthorhombic phases of Cu_2_SnS_3_. Orbital-resolved DOS are provided in Figure S15.

To compare the optoelectronic properties of the monoclinic to the orthorhombic polymorph of Cu_2_SnS_3_, we calculated the band structure and density of states (DOS) for both polymorphs in their lowest-energy relaxed geometries using the HSE06 functional (Figures 2, S14, and 15). Although we did not experimentally refine the monoclinic polymorph, it has previously been determined to be the thermodynamically preferred lowest-energy phase, thus serving as the benchmark to which we compare our proposed orthorhombic polymorph of Cu_2_SnS_3_.^20^ Additionally, the refined tetragonal polymorph possesses a disordered crystal structure (metal site contains 66.7% Cu + 33.3% Sn) resulting in an extensive and impractical site-occupation search to determine an ordered tetragonal polymorph to calculate due to the atomic position occupations being fractional rather than integer values.^20^ To check that the tetragonal polymorph is not significantly more stable than the monoclinic and orthorhombic polymorphs, we have sampled several tetragonal structures and calculated their energies. The sampled structures had energies of 80–90 meV atom^–1^, >3 *k*_B_*T* for *T* = 300 K, higher than the monoclinic structure and an order of magnitude higher than the orthorhombic polymorph. The valence band maxima (VBM) are composed primarily of Cu-d and S-p orbitals for both polymorphs. The most considerable contributions to the conduction band minima (CBM) originate from S-s and p and Sn-s for both polymorphs (Figure S15). Interestingly, while both polymorphs have a low DOS near the CBM, the DOS is significantly lower for the orthorhombic polymorph of Cu_2_SnS_3_. A dense sampling of the Brillouin zone is required to obtain an accurate band structure along its high-symmetry *k*-path. We employed the computationally expensive and accurate HSE06 DFT functional for this purpose (Figure 2). Both polymorphs feature a steep dispersion of the CBM around the Γ point, which rationalizes the lower DOS near the CBM. The orthorhombic polymorph may be preferable over the monoclinic polymorph in solar absorber applications because it has fewer states and lower DOS near the CBM, as can be seen in the larger-scale DOS shown in Figures S14 and S15. Sparse manifolds of states near the CBM can increase the lifetime of hot electrons.^67^ The direct Γ–Γ band gaps of Cu_2_SnS_3_, as determined by the DOS and band structure calculations, are 0.64 and 0.49 eV for orthorhombic and monoclinic polymorphs, respectively. The predicted direct band gap nature of orthorhombic Cu_2_SnS_3_ from the calculations agrees with the experiment; however, the calculations underestimate these values likely due to the strong electron correlation in these materials that may not be fully captured even by the HSE06 functional.

### Property Measurements

**Figure 3:**
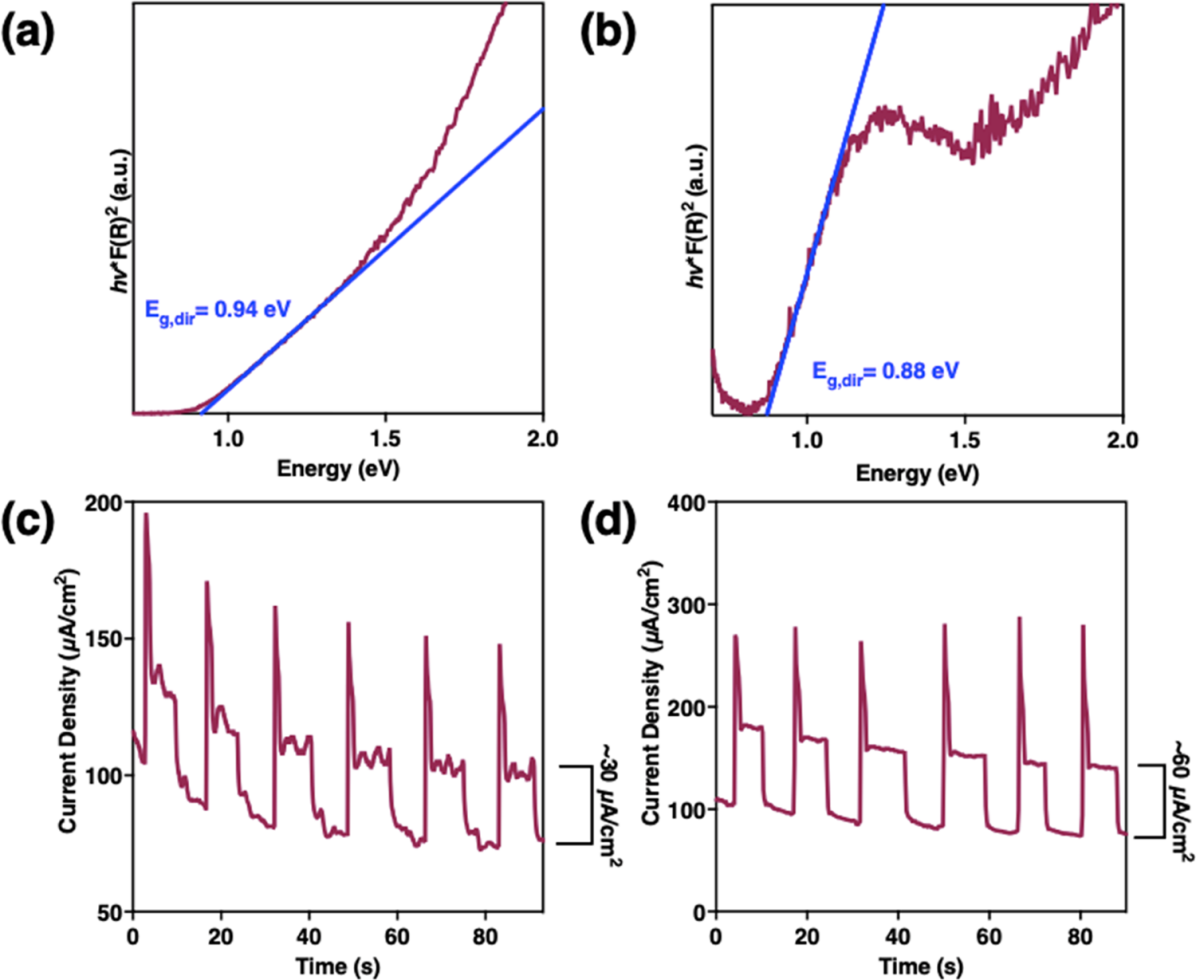
Kubelka–Munk functions of absorption data to estimate direct optical band gaps for (a) tetragonal and (b) orthorhombic polymorphs of Cu_2_SnS_3_. Transient photocurrent response of solution-processed (c) tetragonal and (d) orthorhombic films of Cu_2_SnS_3_ deposited on FTO substrates in 0.1 M Na_2_S/0.01 M sulfur (aq) electrolyte under a potential of −600 mV vs Pt pseudoreference electrode using chopped AM1.5 light.

The optical band gap of the resulting tetragonal and orthorhombic polymorphs were measured by UV–vis–NIR spectroscopy (Figures 3a,b, S16, and S17). Similar direct optical band gaps of *E*_g,dir_ = 0.94 and 0.88 eV for the tetragonal and orthorhombic polymorphs of Cu_2_SnS_3_, respectively, were determined by extrapolating the square of the linear portion of the Kubelka–Munk function-treated diffuse reflectance spectra. These band gaps lie within the range of previously reported experimental values for both tetragonal Cu_2_SnS_3_ and “wurtzite” Cu_2_SnS_3_ nanocrystals.^1,3,21,61^ To demonstrate the photoresponse of these films, transient electrochemical photocurrent response measurements were performed with the two nanostructured polymorphs drop-cast on FTO. The measurements were performed in an electrolyte solution of 0.1 M Na_2_S/0.01 M sulfur dissolved in deionized water with Pt-wire counter and pseudoreference electrodes and the Cu_2_SnS_3_/FTO as the working electrode. With chopped illumination from a standard laboratory solar simulator, controlled potential electrolysis performed with an applied potential of −600 mV yielded stable photocurrents of ∼30 and ∼60 μA cm^–2^ for the tetragonal and orthorhombic phases, respectively (Figure 3c,d). The same measurements performed at positive applied potentials did not return a photocurrent, which confirms the p-type nature of these materials.^3−6^

### Influence of Ink Formulation on Polymorph

**Figure 4:**
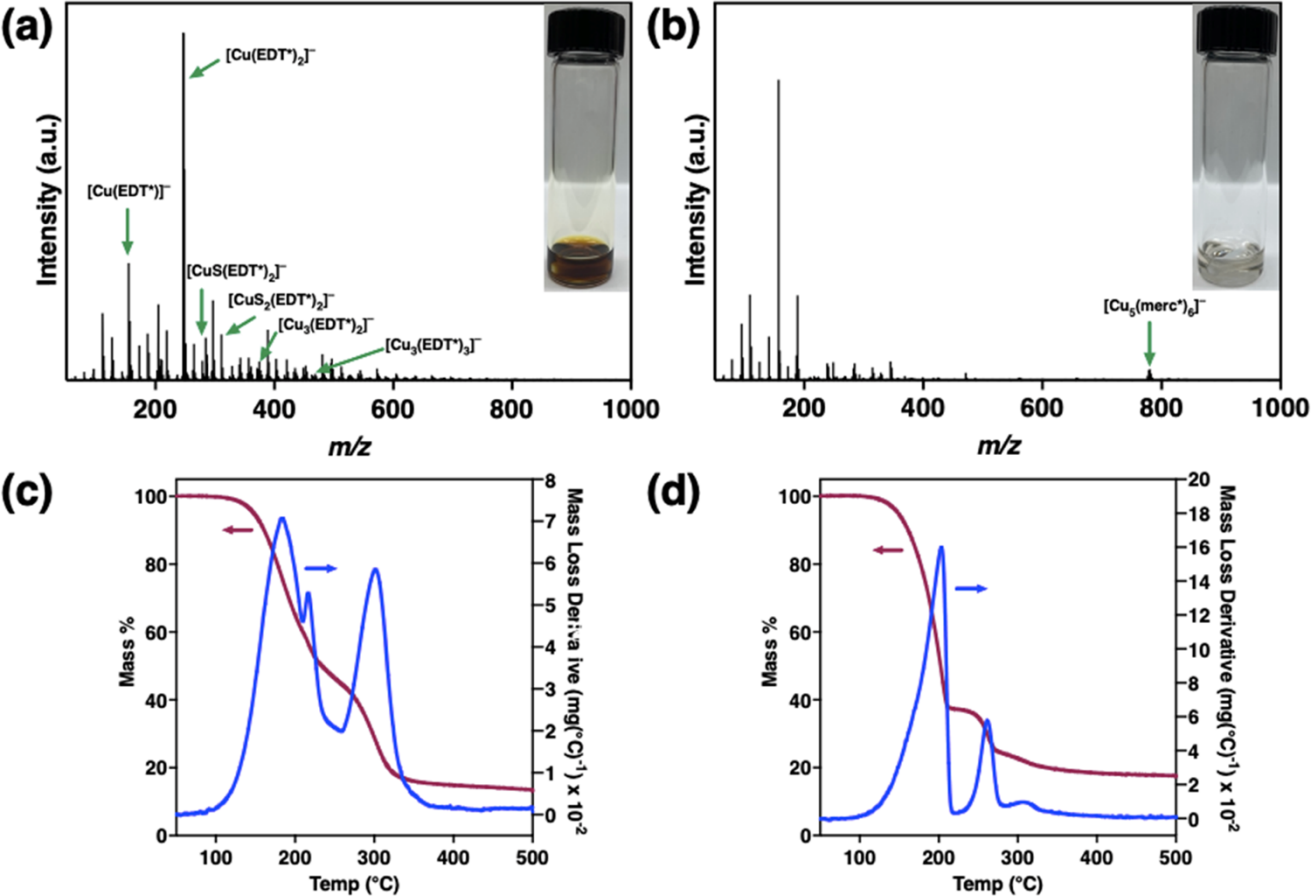
ESI-(−)MS of Cu_2_S dissolved in (a) EDT/en and (b) merc/en. TGA traces and derivative curves of a dried (c) Cu_2_S EDT/en ink and (d) Cu_2_S merc/en ink.

It has been well demonstrated that metastable, wurtzite-like polymorphs of multinary copper-containing chalcogenide nanocrystals are often templated by the initial nucleation of binary copper chalcogenide seeds with hexagonal (or nearly hexagonal) S^2–^ or Se^2–^ sublattices.^68−70^ Indeed, this has also been observed in colloidal Cu_2_SnS_3_ nanocrystals, where “wurtzite” Cu_2_SnS_3_ was thought to initially nucleate as Cu_2–*x*_S with a hexagonal close-packed anion sublattice.^71^ To better understand the influence of ink formulation on Cu_2_SnS_3_ polymorph determination here, we employed a combination of TGA, negative ion mode electrospray ionization mass spectrometry (ESI-(−)MS), and powder XRD to study the differences between the Cu_2_S inks dissolved in EDT/en or merc/en. While ESI-(−)MS is an indirect method, it is effective in gaining insights into the identities of possible molecular solutes formed in thiol–amine inks.^29,72,73^ TGA has been utilized to monitor decomposition temperatures of metal thiolates, which are known to decompose over a wide temperature range (100–350 °C) depending on the identity of the metal (e.g., Cu, Sn, In, etc.) and thiol used.^74−78^ Photographs of the Cu_2_S inks in EDT/en and merc/en are supplied as insets of Figure 4a,b, showing major color differences between the two, with the EDT/en ink being dark orange/brown and the merc/en ink being virtually colorless. Direct-injection ESI-(−)MS was conducted to gain insights into the differences in the molecular solutes resulting from Cu_2_S dissolution in EDT/en and merc/en. The ESI-(−)MS data in Figure 4a,b reveal distinct differences in the major ions between the Cu_2_S EDT/en and merc/en inks. In the EDT/en ink, the major ion at *m*/*z* 246.8810 was attributed to [Cu(C_2_H_4_S_2_)_2_]^−^ (or [Cu(EDT*)_2_]^−^), with the rest of the major ions attributed to different copper thiolate complexes. In the merc/en ink, the major ion at *m*/*z* 776.6852 was attributed to the polynuclear copper cluster [Cu_5_(C_2_H_5_OS)_6_]^−^ (or [Cu_5_(merc*)_6_]^−^).

Derivative curves of TGA traces of these dried Cu_2_S inks with EDT/en and merc/en show differences between the high-temperature mass loss events (Figure 4c,d). The high-temperature mass loss event is larger in the EDT/en ink (27%) and occurs at higher temperatures (305 °C) relative to the merc/en ink (12% mass loss at 264 °C). The lower %mass loss suggests that [Cu_5_(merc*)_6_]^−^ decomposition in the merc/en ink begins well below the high-temperature mass loss event at 264 °C, given the identical ink concentrations. EDT is expected to bind more strongly to copper as a bidentate X_2_-type ligand, whereas merc is expected to bind less strongly in either a bidentate or monodentate fashion as an XL-type or X-type ligand, respectively. Thus, the observed differences in decomposition profiles are consistent with the coordination chemistry of the EDT and merc.

Given the differences in the Cu_2_S inks in terms of their color, solute identity, and decomposition profile, we next sought to understand if the two inks crystallized in different phases of Cu_2–*x*_S. The powder XRD patterns of the Cu_2_S inks dissolved in EDT/en and merc/en and annealed to 235 °C are given in Figure S18. This temperature was chosen due to this being the end of the first major mass loss event for both inks. It is observed that the EDT/en ink results in a cubic Cu_2–*x*_S phase with a cubic close-packed anion sublattice (Figure S18a), which ultimately leads to tetragonal Cu_2_SnS_3_. On the other hand, the merc/en ink results in the formation of a monoclinic Cu_2–*x*_S phase with a hexagonal close-packed anion sublattice (Figure S18b), with ultimately leads to orthorhombic Cu_2_SnS_3_. Taken in concert, we hypothesize that the binding strength of the metal thiolates between the two inks leads to the polymorphic control of our Cu_2_SnS_3_ films. The lower-temperature decomposition of the merc/en ink resulting from a less thermally stable [Cu_5_(merc*)_6_]^−^ complex is a likely kinetic template for the growth of metastable wurtzite-like Cu_2_SnS_3_ through a transient monoclinic Cu_2–*x*_S phase. The [Cu(EDT*)_2_]^−^ complexes formed in the EDT/en inks decompose at higher temperatures due to the more tightly bound EDT ligands, forming the tetragonal polymorph of Cu_2_SnS_3_ from Cu_2–*x*_S with a cubic S^2–^ sublattice.

## Conclusions

In summary, we have demonstrated the ability of an alkahest solvent system to allow for polymorph control in solution-processed tetragonal and orthorhombic Cu_2_SnS_3_ films by simply switching the identity of the thiol. When employing EDT/en as the solvents, the thermodynamically preferred tetragonal phase is recovered upon mild annealing. When the identity of the thiol is switched to merc, a metastable wurtzite-like orthorhombic polymorph is recovered. The solution-processed polymorph control is due to the relative strengths of the resultant metal–thiolate complexes formed in solution, which dictates the decomposition profiles and anionic sublattices of the binary Cu_2–*x*_S phases that likely template the final Cu_2_SnS_3_ polymorphs, as has been described with numerous colloidal nanocrystal-based systems. The p-type tetragonal and orthorhombic films possess experimentally determined direct optical band gaps of 0.94 and 0.88 eV, respectively, and strong photocurrent responses. Understanding how the two ink formulations dictate polymorph crystallization should inform the decomposition and subsequent polymorph control of solution-processed thin films from other thiol–amine inks, such as possible polymorph control in quaternary materials such as Cu_2_ZnSnS_4_. DFT calculations reveal that the wurtzite-like orthorhombic polymorph has a favorable electronic structure compared to the thermodynamic monoclinic polymorph due to a lower DOS near the CBM suggesting increased hot carrier lifetimes beneficial for solar absorbers or other optoelectronic applications.

## Supporting Information Available

Ternary and binary ink TGA/FT-IR; Rietveld refinement plots; refinement statistics of annealed powders; SEM micrographs; UV–vis spectra; XPS survey and high-resolution spectra; Raman spectra of annealed films; and XRD patterns of annealed Cu_2_S inks (PDF)

## References

[ref1] Liu X.; Wang X.; Swihart M. T. Composition-Dependent Crystal Phase, Optical Properties, and Self-Assembly of Cu–Sn–S Colloidal Nanocrystals. Chem. Mater. 2015, 27, 1342–1348. 10.1021/cm504411a.

[ref2] Kolny-Olesiak J.; Weller H. Synthesis and Application of Colloidal CuInS_2_ Semiconductor Nanocrystals. ACS Appl. Mater. Interfaces 2013, 5, 12221–12237. 10.1021/am404084d. 24187935

[ref3] Ghorpade U. V.; Suryawanshi M. P.; Shin S. W.; Kim I.; Ahn S. K.; Yun J. H.; Jeong C.; Kolekar S. S.; Kim J. H. Colloidal Wurtzite Cu_2_SnS_3_ (CTS) Nanocrystals and Their Applications in Solar Cells. Chem. Mater. 2016, 28, 3308–3317. 10.1021/acs.chemmater.6b00176.

[ref4] Kanai A.; Toyonaga K.; Chino K.; Katagiri H.; Araki H. Fabrication of Cu_2_SnS_3_ Thin-Film Solar Cells with Power Conversion Efficiency of over 4%. Jpn. J. Appl. Phys. 2015, 54, 08KC06 10.7567/JJAP.54.08KC06.

[ref5] Berg D. M.; Djemour R.; Gutay L.; Zoppi G.; Siebentritt S.; Dale P. J. Thin Film Solar Cells based on the Ternary Compound Cu_2_SnS_3_. Thin Solid Films 2012, 520, 6291–6294. 10.1016/j.tsf.2012.05.085.

[ref6] Avellaneda D.; Nair M. T.; Nair P. K. Cu_2_SnS_3_ and Cu_4_SnS_4_ Thin Films via Chemical Deposition for Photovoltaic Application. J. Electrochem. Soc. 2010, 157, D346 10.1149/1.3384660.

[ref7] Lee J. Y.; Kim I. Y.; Surywanshi M. P.; Ghorpade U. V.; Lee D. S.; Kim J. H. Fabrication of Cu_2_SnS_3_ Thin Film Solar Cells Using Cu/Sn Layered Metallic Precursors Prepared by a Sputtering Process. Sol. Energy 2017, 145, 27–32. 10.1016/j.solener.2016.09.041.

[ref8] Suryawanshi M. P.; Ghorpade U. V.; Shin S. W.; Pawar S. A.; Kim I. Y.; Hong C. W.; Wu M.; Patil P. S.; Moholkar A. V.; Kim J. H. A Simple Aqueous Precursor Solution Processing of Earth-Abundant Cu_2_SnS_3_ Absorbers for Thin-Film Solar Cells. ACS Appl. Mater. Interfaces 2016, 8, 11603–11614. 10.1021/acsami.6b02167. 27105056

[ref9] Li J.; Xue C.; Wang Y.; Jiang G.; Liu W.; Zhu C. Cu_2_SnS_3_ Solar Cells Fabricated by Chemical Bath Deposition–Annealing of SnS/Cu Stacked Layers. Sol. Energy Mater. Sol. Cells 2016, 144, 281–288. 10.1016/j.solmat.2015.09.017.

[ref10] Tiwari D.; Chaudhuri T. K.; Shripathi T.; Deshpande U.; Rawat R. Non-Toxic, Earth-Abundant 2% Efficient Cu_2_SnS_3_ Solar Cell Based on Tetragonal Films Direct-Coated from Single Metal-Organic Precursor Solution. Sol. Energy Mater. Sol. Cells 2013, 113, 165–170. 10.1016/j.solmat.2013.02.017.

[ref11] Marquez Prieto J. A.; Levcenko S.; Just J.; Hampel H.; Forbes I.; Pearsall N. M.; Unold T. Earth Abundant Thin Film Solar Cells from Co-Evaporated Cu_2_SnS_3_ Absorber Layers. J. Alloys Compd. 2016, 689, 182–186. 10.1016/j.jallcom.2016.07.293.

[ref12] Vadivel S.; Maruthamani D.; Paul B.; Dhar S. S.; Habibi-Yangjeh A.; Balachandran S.; Saravanakumar B.; Selvakumar A.; Selvam K. Biomolecule-Assisted Solvothermal Synthesis of Cu_2_SnS_3_ Flowers/RGO Nanocomposites and Their Visible-Light-Driven Photocatalytic Activities. RSC Adv. 2016, 6, 74177–74185. 10.1039/C6RA12068G.

[ref13] Guo Y.; Yin X.; Yang Y.; Que W. Construction of ZnO/Cu_2_SnS_3_ Nanorod Array Films for Enhanced Photoelectrochemical and Photocatalytic Activity. RSC Adv. 2016, 6, 104041–104048. 10.1039/C6RA22674D.

[ref14] Sun W.; Ye Y.; You Y.; Xu J. A Top-Down Synthesis of Wurtzite Cu_2_SnS_3_ Nanocrystals for Efficient Photoelectrochemical Performance. J. Mater. Chem. A 2018, 6, 8221–8226. 10.1039/C8TA00851E.

[ref15] Xu J.; Yang X.; Wong T.-L.; Lee C.-S. Large-Scale Synthesis of Cu_2_SnS_3_ and Cu_1.8_S Hierarchical Microspheres as Efficient Counter Electrode Materials for Quantum Dot Sensitized Solar Cells. Nanoscale 2012, 4, 6537–6542. 10.1039/c2nr31724a. 22968176

[ref16] Liu F.; Hu S.; Ding X.; Zhu J.; Wen J.; Pan X.; Chen S.; Nazeeruddindm M. K.; Dai S. Ligand-Free Nano-Grain Cu_2_SnS_3_ as a Potential Cathode Alternative for Both Cobalt and Iodine Redox Electrolyte Dye-Sensitized Solar Cells. J. Mater. Chem. A 2016, 4, 14865–14876. 10.1039/C6TA05871J.

[ref17] Lohani K.; Nautiyal H.; Ataollahi N.; Maji K.; Guilmeau E.; Scardi P. Effects of Grain Size on the Thermoelectric Properties of Cu_2_SnS_3_: An Experimental and First-Principles Study. ACS Appl. Energy Mater. 2021, 4, 12604–12612. 10.1021/acsaem.1c02377.

[ref18] Lohani K.; Nautiyal H.; Ataollahi N.; Fanciulli C.; Serguuv I.; Etter M.; Scardi P. Experimental and *Ab Initio* Study of Cu_2_SnS_3_ (CTS) Polymorphs for Thermoelectric Applications. J. Phys. Chem. C 2021, 125, 178–188. 10.1021/acs.jpcc.0c09139.

[ref19] Fiechter S.; Martinez M.; Schmidt G.; Henrion W.; Tomm Y. Phase Relations and Optical Properties of Semiconducting Ternary Sulfides in the System Cu–Sn–S. J. Phys. Chem. Solids 2003, 64, 1859–1862. 10.1016/S0022-3697(03)00172-0.

[ref20] Zhai Y.-T.; Chen S.; Yang J.-H.; Xiang H.-J.; Gong X.-G.; Walsh A.; Kang J.; Wei S.-H. Structural Diversity and Electronic Properties of Cu_2_Sn*X*_3_ (*X* = S, Se): A First-Principles Investigation. Phys. Rev. B 2011, 84, 075213 10.1103/PhysRevB.84.075213.

[ref21] Liu Q.; Zhao Z.; Lin Y.; Guo P.; Li S.; Pan D.; Ji X. Alloyed (ZnS)_*x*_(Cu_2_SnS_3_)_1–*x*_ and (CuInS_2_)_*x*_(Cu_2_SnS_3_)_1–*x*_ Nanocrystals with Arbitrary Composition and Broad Tunable Band Gaps. Chem. Commun. 2011, 47, 964–966. 10.1039/C0CC03560B. 21079830

[ref22] Fan F.-J.; Wu L.; Gong M.; Liu G.; Wang Y.-X.; Yu S.-H.; Chen S.; Wang L.-W.; Gong X.-G. Composition and Band Gap Tunable Synthesis of Wurtzite-Derived Cu_2_ZnSn(S_1–*x*_Se_*x*_)_4_ Nanocrystals: Theoretical and Experimental Insights. ACS Nano 2013, 7, 1454–1463. 10.1021/nn3052296. 23350525

[ref23] Singh A.; Singh S.; Levcenko S.; Unold T.; Laffir F.; Ryan K. M. Compositionally Tunable Photoluminescence Emission in Cu_2_ZnSn(S_1–*x*_Se_*x*_)_4_ Nanocrystals. Angew. Chem., Int. Ed. 2013, 52, 9120–9124. 10.1002/anie.201302867. 23780738

[ref24] Zhang X.; Guo G.; Ji C.; Huang K.; Zha C.; Wang Y.; Shen L.; Gupta A.; Bao N. Efficient Thermolysis Route to Monodisperse Cu_2_ZnSnS_4_ Nanocrystals with Controlled Shape and Structure. Sci. Rep. 2015, 4, 5086 10.1038/srep05086. PMC5381502 24866987

[ref25] Tappan B. A.; Brutchey R. L. Polymorphic Metastability in Colloidal Semiconductor Nanocrystals. ChemNanoMat 2020, 6, 1567–1588. 10.1002/cnma.202000406.

[ref26] Kovalenko M. V.; Scheele V.; Talapin D. V. Colloidal Nanocrystals with Molecular Metal Chalcogenide Surface Ligands. Science 2009, 324, 1417–1420. 10.1126/science.1170524. 19520953

[ref27] Tang J.; Kemp K. W.; Hoogland S.; Jeong K. S.; Liu H.; Levina L.; Furukawa M.; Wang X. H.; Debnath R.; Cha D. K.; Chou K. W.; Fischer A.; Amassian A.; Asbury J. B.; Sargent E. H. Colloidal-Quantum-Dot Photovoltaic using Ligand Passivation. Nat. Mater. 2011, 10, 765–771. 10.1038/nmat3118. 21927006

[ref28] Webber D. H.; Brutchey R. L. Alkahest V_2_VI_3_ Chalcogenides: Dissolution of Nine Semiconductors in a Diamine–Dithiol Solvent Mixture. J. Am. Chem. Soc. 2013, 135, 15722–15725. 10.1021/ja4084336. 24125431

[ref29] Koskela K. M.; Strumolo M. J.; Brutchey R. L. Progress of Thiol-Amine ‘Alkahest’ Solutions for Thin Film Deposition. Trends Chem. 2021, 3, 1061–1073. 10.1016/j.trechm.2021.09.006.

[ref30] Zhao Y.; Yuan S.; Chang Q.; Zhou Z.; Kou D.; Zhou W.; Qi Y.; Wu S. Controllable Formation of Ordered Vacancy Compound for High Efficiency Solution Processed Cu(In,Ga)Se_2_ Solar Cells. Adv. Funct. Mater. 2021, 31, 2007928 10.1002/adfm.202007928.

[ref31] Zhao Y.; Zhao X.; Kou D.; Zhou W.; Zhou Z.; Yuan S.; Qi Y.; Zheng Z.; Wu S. Local Cu Component Engineering to Achieve Continuous Carrier Transport for Enhanced Kesterite Solar Cells. ACS Appl. Mater. Interfaces 2021, 13, 795–805. 10.1021/acsami.0c21008. 33397088

[ref32] McCarthy C. L.; Downes C. A.; Schueller E. C.; Abuyen K.; Brutchey R. L. Method for the Solution Deposition of Phase-Pure CoSe_2_ as an Efficient Hydrogen Evolution Reaction Electrocatalyst. ACS Energy Lett. 2016, 1, 607–611. 10.1021/acsenergylett.6b00246.

[ref33] McCarthy C. L.; Downes C. A.; Brutchey R. L. Room Temperature Dissolution of Bulk Elemental Ni and Se for Solution Deposition of a NiSe_2_ HER Electrocatalyst. Inorg. Chem. 2017, 56, 10143–10146. 10.1021/acs.inorgchem.7b01594. 28816446

[ref34] Liu Y.; Calcabrini M.; Yu Y.; Lee S.; Chang C.; David J.; Ghosh T.; Spadaro M. C.; Xie C.; Cojocaru- Mirédin O.; Arbiol J.; Ibáñez M. Defect Engineering in Solution–Processed Polycrystalline SnSe Leads to High Thermoelectric Performance. ACS Nano 2022, 16, 78–88. 10.1021/acsnano.1c06720. PMC8793148 34549956

[ref35] Chang C.; Liu Y.; Lee S. H.; Spadaro M. C.; Koskela K. M.; Kleinhanns T.; Costanzo T.; Arbiol J.; Brutchey R. L.; Ibáñez M. Surface Functionalization of Surfactant-Free Particles: A Strategy to Tailor the Properties of Nanocomposites for Enhanced Thermoelectric Performance. Angew. Chem., Int. Ed. 2022, 61, e202207002 10.1002/anie.202207002. PMC9542085 35799379

[ref36] Zuo Y.; Li J.; Yu X.; Du R.; Zhang T.; Wang X.; Arbiol J.; Llorca J.; Cabot A. A SnS_2_ Molecular Precursor for Conformal Nanostructured Coatings. Chem. Mater. 2020, 32, 2097–2106. 10.1021/acs.chemmater.9b05241.

[ref37] Hasan M. R.; Arinze E. S.; Singh A. K.; Oleshko V. P.; Guo S.; Rani A.; Cheng Y.; Kalish I.; Zaghloul M. E.; Rao M. V. An Antimony Selenide Molecular Ink for Flexible Broadband Detectors. Adv. Electron. Mater. 2016, 2, 1600182 10.1002/aelm.201600182. 27840807 PMC5103318

[ref38] McCarthy C. L.; Brutchey R. L. Solution Deposited Cu_2_BaSnS_4-*x*_Se_*x*_ from a Thiol–Amine Solvent Mixture. Chem. Mater. 2018, 30, 304–308. 10.1021/acs.chemmater.7b03931.

[ref39] Koskela K. M.; Melot B. C.; Brutchey R. L. Solution Deposition of a Bournonite CuPbSbS_3_ Semiconductor Thin Film from the Dissolution of Bulk Materials with a Thiol–Amine Solvent Mixture. J. Am. Chem. Soc. 2020, 142, 6173–6179. 10.1021/jacs.9b13787. 32160454

[ref40] Nagel A.; Range K. J. Verbindungsbildung im System Ag2S-GeS2-AgI/Compound Formation in the System Ag2S-GeS2-AgI. Z. Naturforsch., B 1978, 33, 1461–1464. 10.1515/znb-1978-1218.

[ref41] Hohenberg P.; Kohn W. Inhomogeneous Electron Gas. Phys. Rev. 1964, 136, B864–B871. 10.1103/PhysRev.136.B864.

[ref42] Kohn W.; Sham L. J. Self-Consistent Equations Including Exchange and Correlation Effects. Phys. Rev. 1965, 140, A1133–A1138. 10.1103/PhysRev.140.A1133.

[ref43] Kohn W. Nobel Lecture: Electronic Structure of Matter–Wave Functions and Density Functionals. Rev. Mod. Phys. 1999, 71, 1253–1266. 10.1103/RevModPhys.71.1253.

[ref44] Kresse G.; Joubert D. From Ultrasoft Pseudopotentials to the Projector Augmented-Wave Method. Phys. Rev. B 1999, 59, 1758–1775. 10.1103/PhysRevB.59.1758.

[ref45] Blöchl P. E. Projector Augmented-Wave Method. Phys. Rev. B 1994, 50, 17953–17979. 10.1103/PhysRevB.50.17953. 9976227

[ref46] Perdew J. P.; Burke K.; Wang Y. Generalized Gradient Approximation for the Exchange-Correlation Hole of a Many-Electron System. Phys. Rev. B 1996, 54, 16533–16539. 10.1103/PhysRevB.54.16533. 9985776

[ref47] Perdew J. P.; Burke K.; Ernzerhof M. Generalized Gradient Approximation Made Simple. Phys. Rev. Lett. 1996, 77, 3865–3868. 10.1103/PhysRevLett.77.3865. 10062328

[ref48] Krukau A. V.; Vydrov O. A.; Izmaylov A. F.; Scuseria G. E. Influence of the Exchange Screening Parameter on the Performance of Screened Hybrid Functionals. J. Chem. Phys. 2006, 125, 224106 10.1063/1.2404663. 17176133

[ref49] Heyd J.; Scuseria G. E. Assessment and Validation of a Screened Coulomb Hybrid Density Functional. J. Chem. Phys. 2004, 120, 7274–7280. 10.1063/1.1668634. 15267636

[ref50] Heyd J.; Peralta J. E.; Scuseria G. E.; Martin R. L. Energy Band Gaps and Lattice Parameters Evaluated with the Heyd-Scuseria-Ernzerhof Screened Hybrid Functional. J. Chem. Phys. 2005, 123, 174101 10.1063/1.2085170. 16375511

[ref51] Heyd J.; Scuseria G. E.; Ernzerhof M. Hybrid Functionals Based on a Screened Coulomb Potential. J. Chem. Phys. 2003, 118, 8207–8215. 10.1063/1.1564060.

[ref52] Kresse G.; Hafner J. Ab initio Molecular Dynamics for Liquid Metals. Phys. Rev. B 1993, 47, 558–561. 10.1103/PhysRevB.47.558. 10004490

[ref53] Kresse G.; Hafner J. Ab initio Molecular-Dynamics Simulation of the Liquid-Metal--Amorphous-Semiconductor Transition in Germanium. Phys. Rev. B 1994, 49, 14251–14269. 10.1103/PhysRevB.49.14251. 10010505

[ref54] Kresse G.; Furthmüller J. Efficiency of Ab-initio Total Energy Calculations for Metals and Semiconductors using Plane-Wave Basis Set. Comput. Mater. Sci. 1996, 6, 15–50. 10.1016/0927-0256(96)00008-0.

[ref55] Kresse G.; Furthmüller J. Efficient Iterative Schemes for Ab initio Total-Energy Calculations using a Plane-Wave Basis Set. Phys. Rev. B 1996, 54, 11169–11186. 10.1103/PhysRevB.54.11169. 9984901

[ref56] Hayashi M.; Shiro Y.; Oshima T.; Murata H. The Vibrational Assignment, Rotational Isomerism and Force Constants of 1,2- Ethanedithiol. Bull. Chem. Soc. Jpn. 1965, 38, 1734–1740. 10.1246/bcsj.38.1734.

[ref57] Nandy S. K.; Mukherjee D. K.; Roy S. B.; Kastha G. S. Vibrational Spectra and Rotational Isomerism in 2-Mercaptoethanol. Can. J. Chem. 1973, 51, 1139–1141. 10.1139/v73-169.

[ref58] Chen X.-a.; Wada H.; Sato A.; Mieno M. Synthesis, Electrical Conductivity, and Crystal Structure of Cu_4_Sn_7_S_16_ and Structure Refinement of Cu_2_SnS_3_. J. Solid State Chem. 1998, 139, 144–151. 10.1006/jssc.1998.7822.

[ref59] Ng M. T.; Boothroyd C. B.; Vittal J. J. One-Pot Synthesis of New-Phase AgInSe_2_ Nanorods. J. Am. Chem. Soc. 2006, 128, 7118–7119. 10.1021/ja060543u. 16734438

[ref60] Shen X.; Hernandez-Pagan E. A.; Zhou W.; Puzyrev Y. S.; Idrobo J.-C.; Macdonald J. E.; Pennycock S. J.; Pantelides S. T. Interlaced Crystals having a Perfect Bravais Lattice and Complex Chemical Order Revealed by Real-Space Crystallography. Nat. Commun. 2014, 5, 5431 10.1038/ncomms6431. 25394496

[ref61] Tiwari D.; Chaudhuri T. K.; Shripathi T.; Deshpande U. Synthesis of Earth-Abundant Cu_2_SnS_3_ Powder Using Solid State Reaction. J. Phys. Chem. Solids 2014, 75, 410–415. 10.1016/j.jpcs.2013.11.012.

[ref62] Cheng A.-J.; Manno M.; Khare A.; Leighton C.; Campbell S. A.; Aydil E. S. Imaging and Phase Identification of Cu_2_ZnSnS_4_ Thin Films Using Confocal Raman Spectroscopy. J. Vac. Sci. Technol., A 2011, 29, 051203 10.1116/1.3625249.

[ref63] Ge J.; Yan Y. Synthesis and Characterization of Photoelectrochemical and Photovoltaic Cu_2_BaSnS_4_ Thin Films and Solar Cells. J. Mater. Chem. C 2017, 5, 6406–6419. 10.1039/C7TC01678F.

[ref64] Koskela K. M.; Tadle A. C.; Chen K.; Brutchey R. L. Solution Processing Cu_3_BiS_3_ Absorber Layers with a Thiol–Amine Solvent Mixture. ACS Appl. Energy Mater. 2021, 4, 11026–11031. 10.1021/acsaem.1c01962.

[ref65] Aykol M.; Dwaraknath S. S.; Sun W.; Persson K. A. Thermodynamic Limit for Synthesis of Metastable Inorganic Materials. Sci. Adv. 2018, 4, eaaq0148 10.1126/sciadv.aaq0148. 29725618 PMC5930398

[ref66] Sun W.; Dacek S. T.; Ong S. P.; Hautier G.; Jain A.; Richards W. D.; Gamst A. C.; Persson K. A.; Ceder G. The Thermodynamic Scale of Inorganic Crystalline Metastability. Sci. Adv. 2016, 2, e1600225 10.1126/sciadv.1600225. 28138514 PMC5262468

[ref67] Wang T.; Jin L.; Hidalgo J.; Chu W.; Snaider J. M.; Deng S.; Zhu T.; Lai B.; Prezhdo O.; Correa-Baena J.; Huang L. Protecting Hot Carriers by Tuning Hybrid Perovskite Structures with Alkali Cations. Sci. Adv. 2020, 6, eabb1336 10.1126/sciadv.abb1336. 33097534 PMC7608821

[ref68] Tappan B. A.; Chu W.; Mecklenburg M.; Prezhdo O. V.; Brutchey R. L. Discovery of a Wurtzite-like Cu_2_FeSnSe_4_ Semiconductor Nanocrystal Polymorph and Implications for Related CuFeSe_2_ Materials. ACS Nano 2021, 15, 13463–13474. 10.1021/acsnano.1c03974. 34346226

[ref69] Tappan B. A.; Barim G.; Kwok J. C.; Brutchey R. L. Utilizing Diselenide Precursors toward Rationally Controlled Synthesis of Metastable CuInSe_2_ Nanocrystals. Chem. Mater. 2018, 30, 5704–5713. 10.1021/acs.chemmater.8b02205.

[ref70] Tappan B. A.; Crans K. D.; Brutchey R. L. Formation Pathway of Wurtzite-like Cu_2_ZnSnSe_4_ Nanocrystals. Inorg. Chem. 2021, 60, 17178–17185. 10.1021/acs.inorgchem.1c02506. 34735130

[ref71] Liang Q. Phase-Controlled Synthesis of Cu_2_SnS_3_ Nanocrystals: The Effect of Redox Conditions on the Initial Binary Cu_2–*x*_S Nucleation. Eur. J. Inorg. Chem. 2016, 2016, 3634–3640. 10.1002/ejic.201600447.

[ref72] Koskela K. M.; Quiton S. J.; Sharada S. M.; Williams T. J.; Brutchey R. L. Kinetics and Mechanistic Details of Bulk ZnO Dissolution Using a Thiol-Imidazole System. Chem. Sci. 2022, 13, 3208–3215. 10.1039/D1SC06667F. 35414876 PMC8926287

[ref73] Lowe J. C.; Wright L. D.; Eremin D. B.; Burykina J. V.; Martens J.; Plasser F.; Ananikov V. P.; Bowers J. W.; Malkov A. V. Solution Processed CZTS Solar Cells using Amine-Thiol Systems: Understanding the Dissolution Process and Device Fabrication. J. Mater. Chem. C 2020, 8, 10309–10318. 10.1039/D0TC00955E.

[ref74] Zhuang Z.; Lu X.; Peng Q.; Li Y. A Facile “Dispersion–Decomposition” Route to Metal Sulfide Nanocrystals. Chem. - Eur. J. 2011, 17, 10445–10452. 10.1002/chem.201101145. 21915921

[ref75] Heo J.; Kim G.-H.; Jeong J.; Yoon Y. J.; Seo J. H.; Walker B.; Kim J. Y. Clean Thermal Decomposition of Tertiary-Alkyl Metal Thiolates to Metal Sulfides: Environmentally-Benign, Non-Polar Inks for Solution-Processed Chalcopyrite Solar Cells. Sci. Rep. 2016, 6, 36608 10.1038/srep36608. 27827402 PMC5101475

[ref76] Kino T.; Kuzuya T.; Itoh K.; Sumiyama K.; Wakamatsu T.; Ichidate M. Synthesis of Chalcopyrite Nanoparticles via Thermal Decomposition of Metal-Thiolate. Mater. Trans., JIM 2008, 49, 435–438. 10.2320/matertrans.MBW200724.

[ref77] Kuyuza T.; Yamamuro S.; Hihara T.; Sumiyama K. Water-Free Synthesis of Monodisperse Cu_2_S Nanocrystals. Chem. Lett. 2004, 33, 352–353. 10.1246/cl.2004.352.

[ref78] Barone G.; Chaplin T.; Hibbert T. G.; Kana A. T.; Mahon M. F.; Molloy K. C.; Worsley I. D.; Parkin I. P.; Price L. S. Synthesis and Thermal Decomposition Studies of Homo- and Heteroleptic Tin(IV) Thiolates and Dithiocarbamates: Molecular Precursors for Tin Sulfides. J. Chem. Soc., Dalton Trans. 2002, 1085–1092. 10.1039/b108509n.

