# Supplementary material for: Polymorphic Control of Solution-Processed Cu2SnS3 Films with Thiol–Amine Ink Formulation
Source: Chem Mater. 2022 Sep 21;34(19):8654–63. doi: 10.1021/acs.chemmater.2c01612 (PMC9558449; doi:10.1021/acs.chemmater.2c01612)
Supplement: Supplementary file 1 — cm2c01612_si_001.pdf [file cm2c01612_si_001.pdf]

Supporting Information

Polymorphic Control of Solution Processed Cu_2SnS_3 Films with Thiol–Amine Ink Formulation

Kristopher M. Koskela,[1] Carlos Mora Perez ,[1] Dmitry B. Eremin,[1,2], Jake M. Evans,[3] Marissa, J. Strumolo,[1] Nathan S. Lewis,[3] Oleg V. Prezhdo,[1]* and Richard L. Brutchey[1]*

[1]Department of Chemistry, University of Southern California, Los Angeles, CA 90089, United States
[2]The Bridge@USC, University of Southern California, Los Angeles, CA 90089, United States
[3]Division of Chemistry and Chemical Engineering, California Institute of Technology, Pasadena, CA 91125, United States

*Emails: prezhdo@usc.edu, brutchey@usc.edu

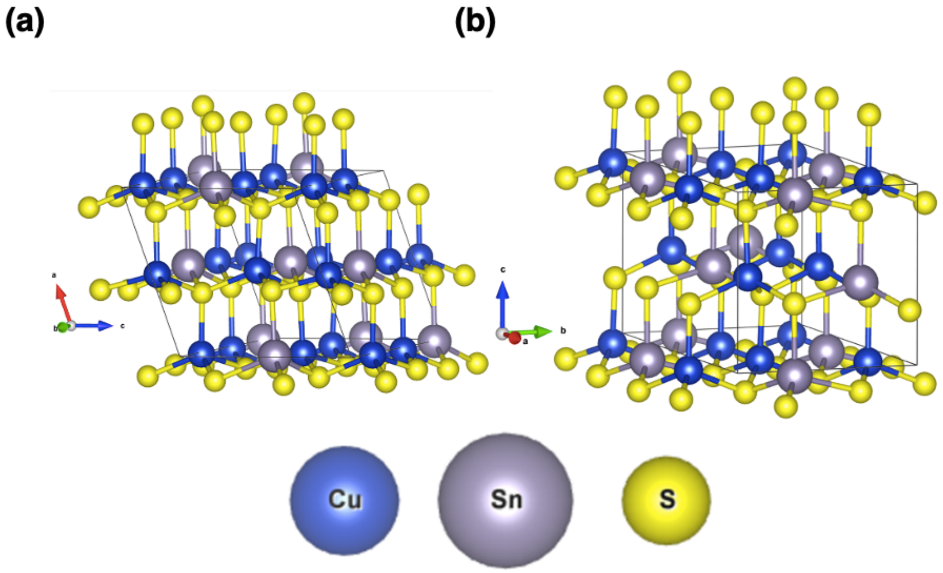

Figure S1. Geometric structures of (a) monoclinic (*Cc*) and (b) orthorhombic (*Cmc*2$_1$) phases, optimized using the PBE functional with the forces converged to 3 meV/Å. Computing the total energies of these structures with the more accurate HSE06 functional indicates that *Cmc*2$_1$ Cu_2SnS_3 has a slightly higher energy than *Cc* Cu_2SnS_3 by 7.6 meV/atom.

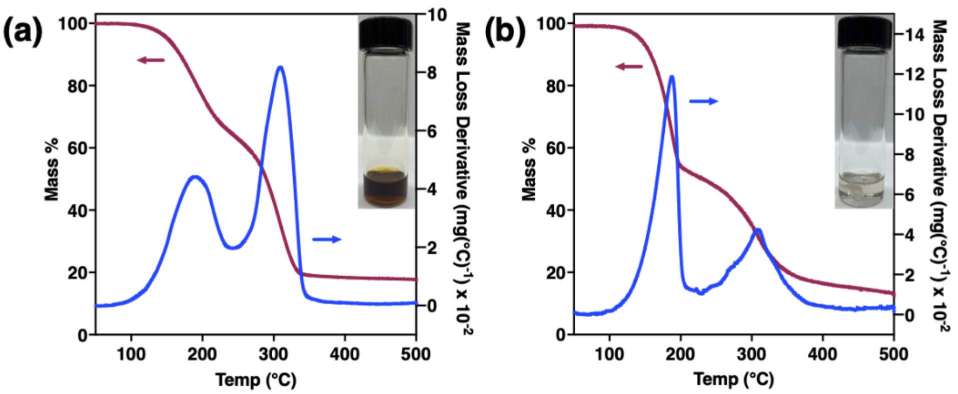

Figure S2. (a) TGA trace and derivative curve of a dried EDT/en ink that yields tetragonal Cu_2SnS_3, demonstrating a decomposition endpoint of < 350 ˚C. Inset is a picture of the ink with Cu_2S and SnO dissolved in EDT/en (1:4 vol/vol). (b) TGA trace and derivative curve of a dried merc/en ink that yields orthorhombic Cu_2SnS_3, demonstrating a decomposition endpoint of < 350 ˚C. Inset is a picture of the combined ink with Cu_2S and SnO dissolved in merc/en (1:4 vol/vol).

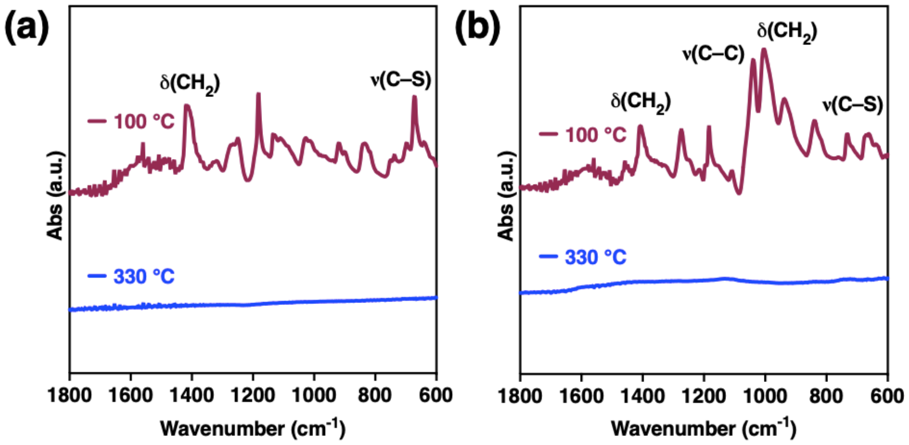

Figure S3. ATR FT-IR spectra of the inks dried at 100 ˚C and annealed to 330 ˚C confirming loss of organic species for (a) tetragonal and (b) orthorhombic Cu_2SnS_3.

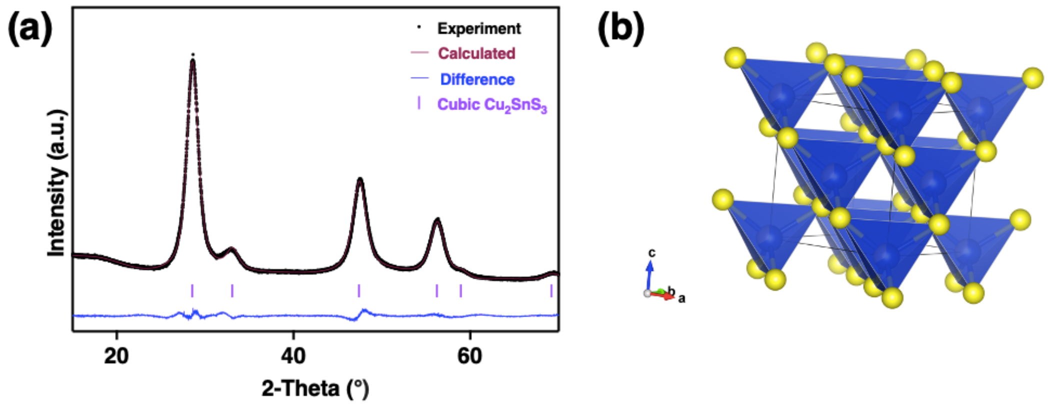

Figure S4. (a) Rietveld refinement of the zinc blende phase using the cubic $F\bar{4}3m$ unit cell (χ^2 3.56, wR 3.32%, a = 5.46 Å). (λ = 1.5406 Å) (b) Structural representation of disordered cubic Cu_2SnS_3. Sulfur atoms are yellow, tin atoms are silver, and copper atoms are blue.

Table S1. Structural parameters of tetragonal ($I\bar{4}2m$) Cu_2SnS_3 extracted from Rietveld analysis.

Space Group	$I\bar{4}2m$
$a = b$ (Å)	5.4267(6)
c (Å)	10.6869(3)
V (Å^3)	314.72(8)
$\alpha = \beta = \gamma$	90°
R_{wp}	2.288%

Atom	Mult.	x	y	z	Frac.	U_{iso}
Sn_1	4	0.00000	0.50000	0.25000	0.1649	0.02270
Cu_1	4	0.00000	0.50000	0.25000	0.2160	0.02322
Sn_2	2	0.00000	0.00000	0.50000	0.1538	0.05255
Cu_2	2	0.00000	0.00000	0.50000	0.1672	0.05684
Cu_3	2	0.00000	0.00000	0.00000	0.4031	0.01006
S_1	8	0.25625	0.25625	0.12715	0.3934	0.03402

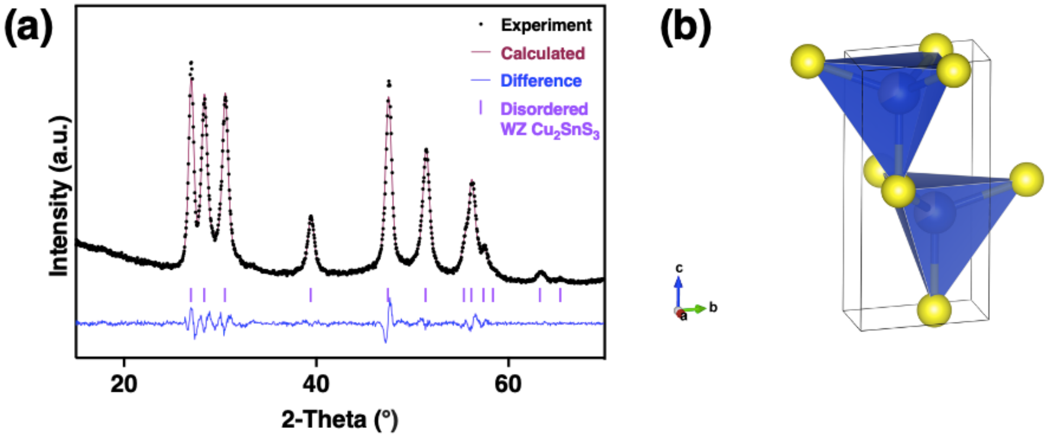

Figure S5. (a) Rietveld refinement of the wurtzite phase using the disordered $P6_3mc$ unit cell (χ^2 3.05, wR 4.754%, a = 3.78 Å, c = 6.44 Å). (λ = 1.5406 Å) (b) Structural representation of disordered wurtzite Cu_2SnS_3.

Table S2. Structural parameters of orthorhombic ($Cmc2_1$) Cu_2SnS_3 extracted from Rietveld analysis.

Space Group	$Cmc2_1$
a (Å)	11.4569(4)
b (Å)	6.6268(9)
c (Å)	6.3215(7)
V (Å^3)	479.95(8)
$\alpha = \beta = \gamma$	90°
R_{wp}	4.018%

Atom	Mult.	x	y	z	Frac.	U_{iso}
Cu_1	8	0.17001	0.83422	0.98810	0.9954	0.01538
S_1	8	0.17757	0.84400	0.35204	1.0417	0.01393
Sn_1	4	0.00000	0.31625	0.96892	0.9898	0.00950
S_2	4	0.00000	0.30922	0.36476	0.9416	0.02614

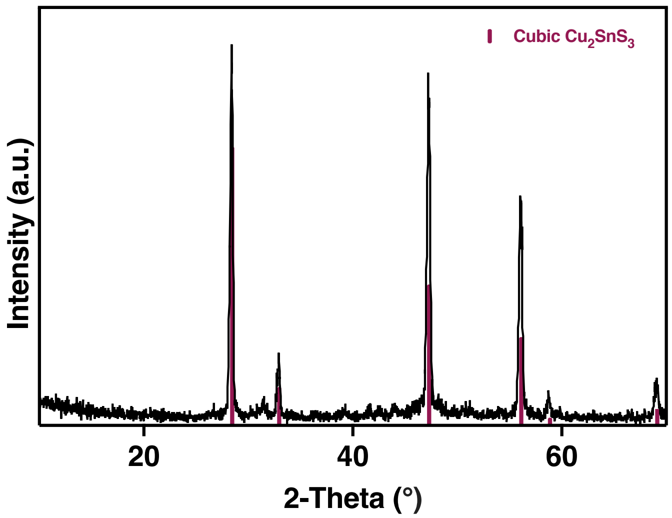

Figure S6. Powder XRD diffraction pattern of the orthorhombic Cu_2SnS_3 polymorph annealed to 550 °C, indexed to a simulated cubic zinc blende structure.

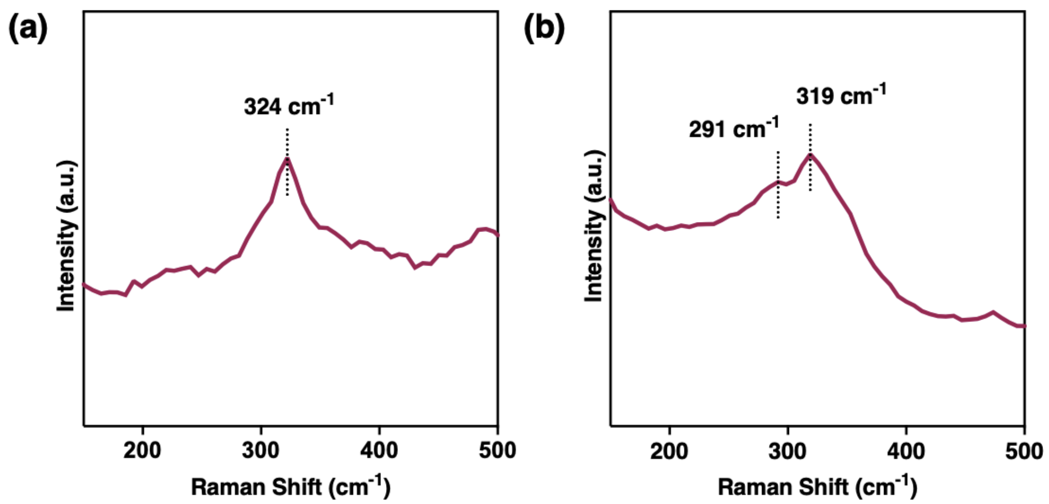

Figure S7. Raman spectra using 532 nm excitation of (a) tetragonal and (b) orthorhombic Cu_2SnS_3 films drop-casted and annealed at 330 °C.

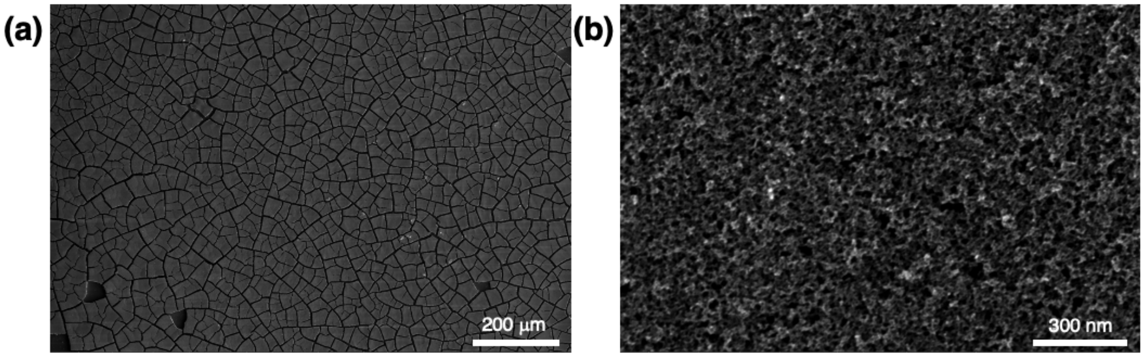

Figure S8. Top-down SEM micrographs of tetragonal Cu_2SnS_3 at (a) 350 and (b) 250,000× drop-casted on Si and annealed at 330 °C.

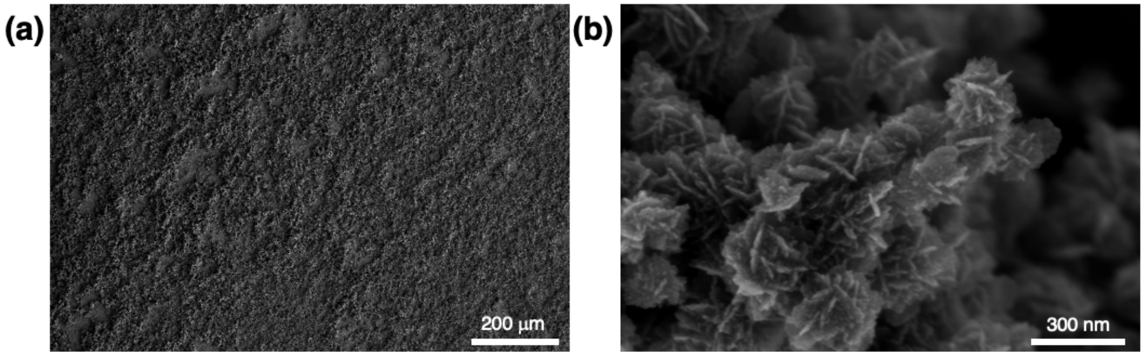

Figure S9. Top-down SEM micrographs of orthorhombic Cu_2SnS_3 at (a) 350 and (b) 250,000× drop-casted on Si and annealed at 330 °C.

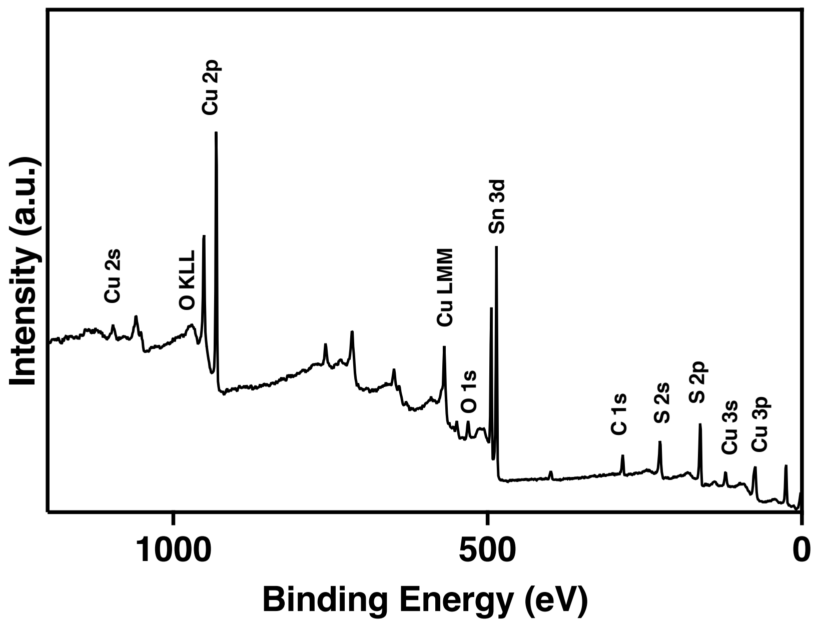

Figure S10. XPS survey scan of tetragonal Cu_2SnS_3 drop casted on Si and annealed to 330 °C.

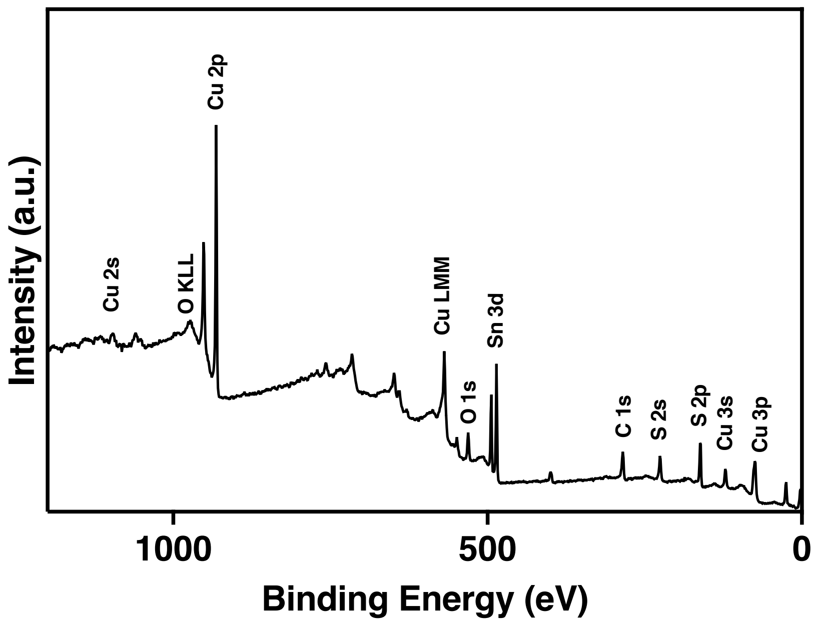

Figure S11. XPS survey scan of orthorhombic Cu_2SnS_3 drop casted on Si and annealed to 330 °C.

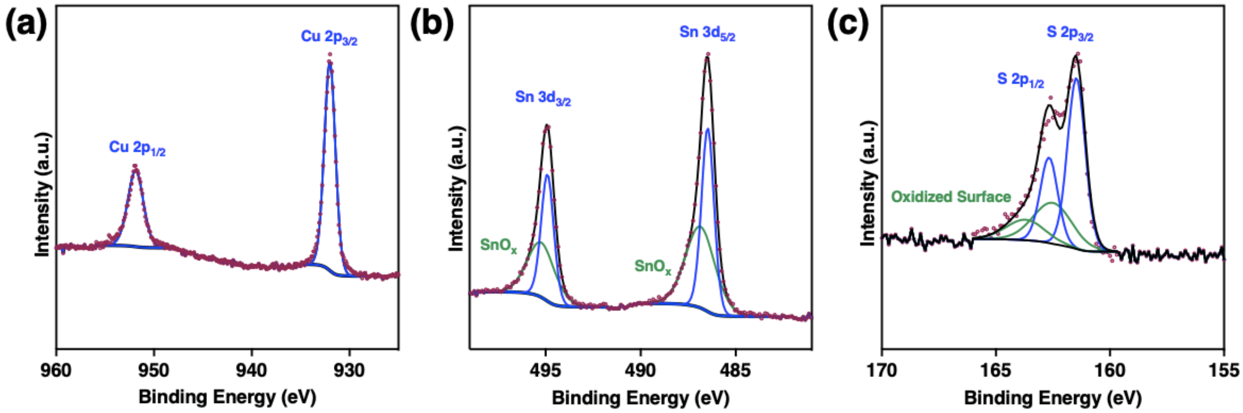

Figure S12. High-resolution XP spectra of (a) Cu 2*p*, (b) Sn 3*d*, and (c) S 2*p* regions of tetragonal Cu_2SnS_3 drop-casted on Si and annealed at 330 °C.

Table S3. Peak positions and peak splitting from the high-resolution XP spectra of tetragonal Cu_2SnS_3 drop-casted on Si and annealed at 330 °C.

Element	Peak Splitting (eV)	Peak ID	Binding Energy (eV)
Cu	19.8	$2p_{1/2}$	952.0
		$2p_{3/2}$	932.2
Sn	8.4	$3d_{3/2}$	494.8
		$3d_{5/2}$	486.4
Sn (surface SnO_x)	8.4	$3d_{3/2}$	495.2
		$3d_{5/2}$	486.8
S	1.2	$2p_{1/2}$	162.6
		$2p_{3/2}$	161.4
S (oxidized surface species)	1.2	$2p_{1/2}$	163.7
		$2p_{3/2}$	162.5

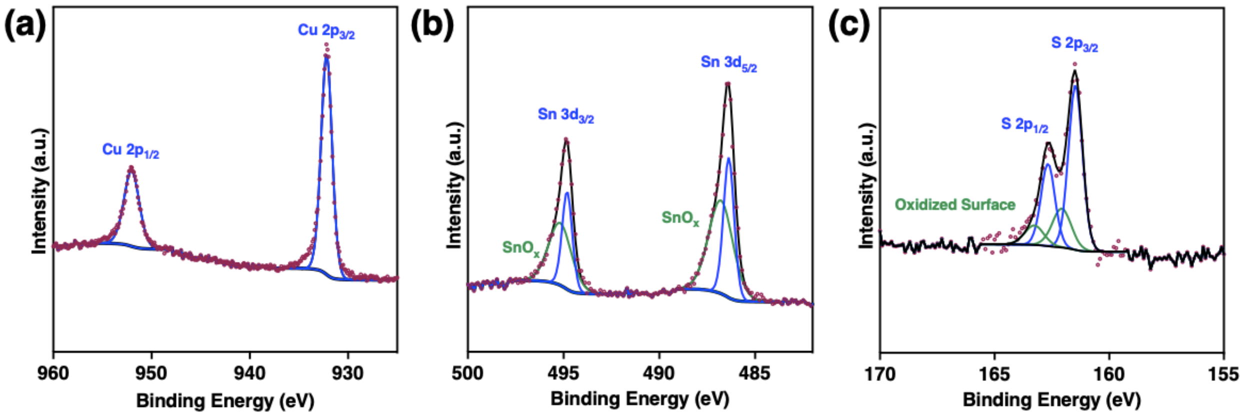

Figure S13. High-resolution XP spectra of (a) Cu 2*p*, (b) Sn 3*d*, and (c) S 2*p* regions of orthorhombic Cu_2SnS_3 drop-casted on Si and annealed at 330 °C.

Table S4. Peak positions and peak splitting from the high-resolution XP spectra of orthorhombic Cu_2SnS_3 drop-casted on Si and annealed at 330 °C.

Element	Peak Splitting (eV)	Peak ID	Binding Energy (eV)
Cu	19.8	$2p_{1/2}$	952.0
		$2p_{3/2}$	932.2
Sn	8.4	$3d_{3/2}$	494.8
		$3d_{5/2}$	486.4
Sn (surface SnO_x)	8.4	$3d_{3/2}$	495.2
		$3d_{5/2}$	486.8
S	1.2	$2p_{1/2}$	162.6
		$2p_{3/2}$	161.4
S (oxidized surface species)	1.2	$2p_{1/2}$	163.2
		$2p_{3/2}$	162.0

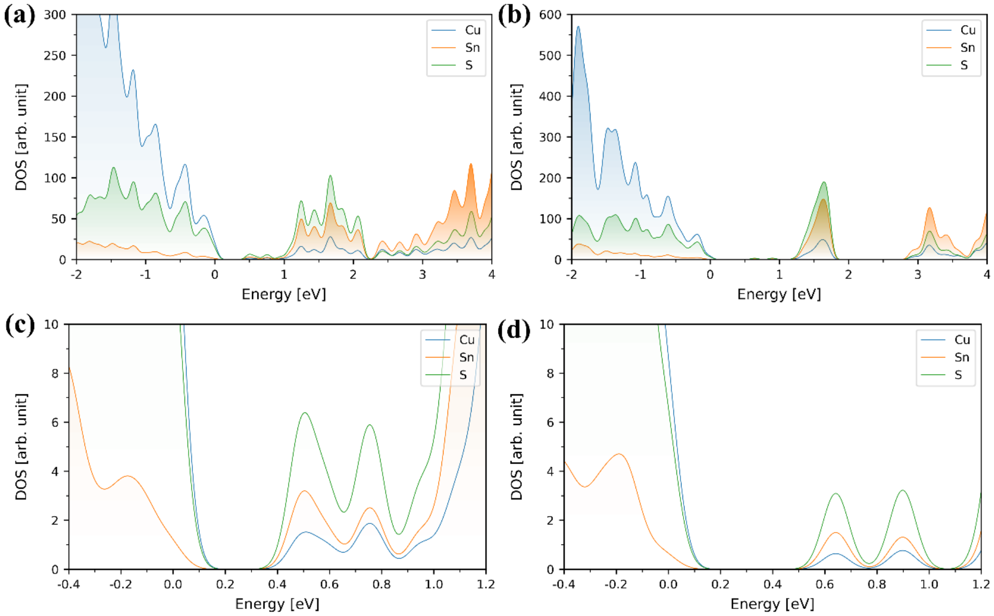

Figure S14. Element projected electronic density of states (DOS) calculated with the HSE06 functional for (a) monoclinic (*Cc*) and (b) orthorhombic (*Cmc*2$_1$) polymorphs of Cu_2SnS_3. DOS near the band edges for (c) monoclinic (*Cc*) and (d) orthorhombic (*Cmc*2$_1$) polymorphs of Cu_2SnS_3.

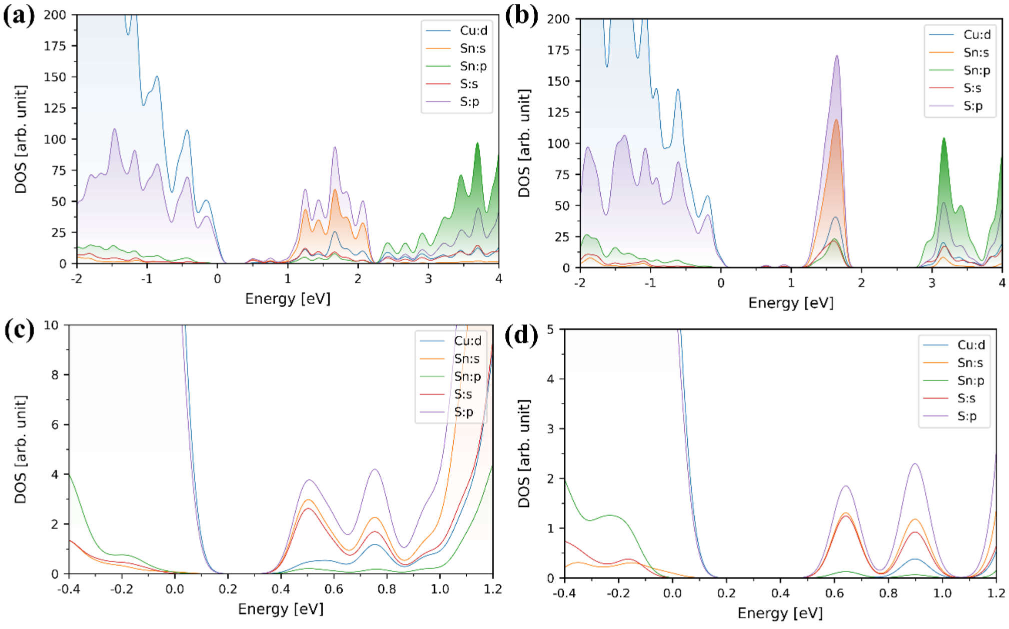

Figure S15. Orbital projected density of states (DOS) calculated with the HSE06 functional for (a) monoclinic (*Cc*) and (b) orthorhombic (*Cmc*2$_1$) polymorphs of Cu_2SnS_3. Orbital projected DOS near the band edges for (c) monoclinic (*Cc*) and (d) orthorhombic (*Cmc*2$_1$) polymorphs of Cu_2SnS_3.

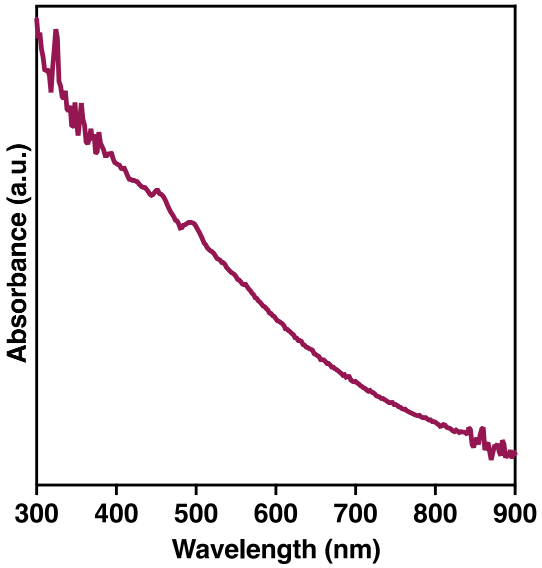

Figure S16. Absorbance spectra of the tetragonal polymorph from 300-900 nm.

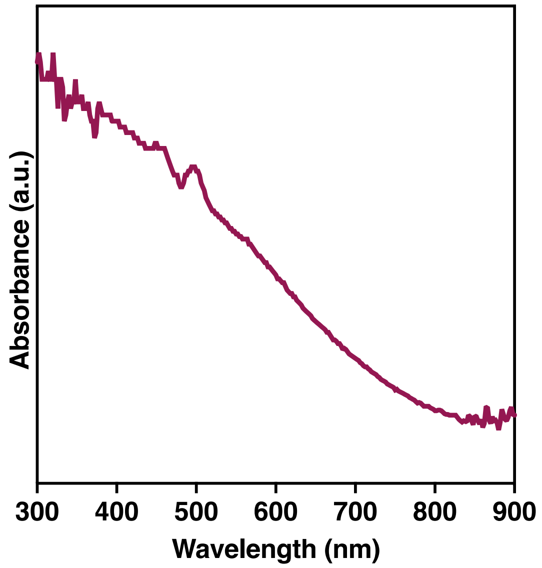

Figure S17. Absorbance spectra of the orthorhombic polymorph from 300-900 nm.

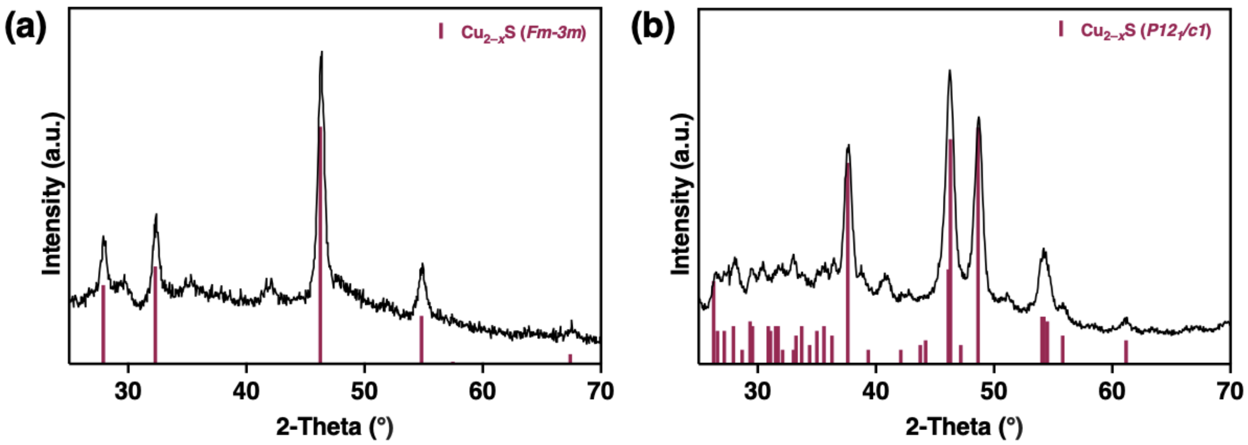

Figure S18. Powder XRD of $Cu_{2-x}S$ resulting from annealing the (a) EDT/en and (b) merc/en inks of Cu_2S to 235 °C. The $Cu_{2-x}S$ resulting from Cu_2S dissolved in EDT/en indexes to cubic $Cu_{2-x}S$ (JCPDS No: 01-073-8624) while the $Cu_{2-x}S$ resulting Cu_2S dissolved in merc/en indexes to monoclinic $Cu_{2-x}S$ (JCPDS No: 00-023-0959), with a hexagonal S^{2-} sublattice.
